# In Vitro Anti-Inflammatory Activity and Molecular Docking Analysis of Compounds Isolated from *Beyeria viscosa*

**DOI:** 10.3390/cimb48050495

**Published:** 2026-05-10

**Authors:** Hamza Shahid, James P. Flood, Feng Li, Xian Zhou, Gerald Münch, Ritesh Raju

**Affiliations:** 1 Department of Pharmacology, School of Medicine, Faculty of Health, Western Sydney University, Campbelltown Campus, Sydney, NSW 2751, Australia; 22113146@student.westernsydney.edu.au (H.S.); g.muench@westernsydney.edu.au (G.M.); 2 School of Science, Faculty of Science, Western Sydney University, Locked Bag 1797, Penrith, NSW 2751, Australia; 19801201@student.westernsydney.edu.au (J.P.F.); feng.li@westernsydney.edu.au (F.L.); 3 National Institute of Complimentary Medicine, Health Research Institute, Western Sydney University, Westmead, NSW 2145, Australia; p.zhou@westernsydney.edu.au

**Keywords:** Australian indigenous plant, anti-inflammatory activity, RAW 264.7 macrophages, N-11 microglial cells, fritillebic acid, X-ray crystallography, molecular docking

## Abstract

Inflammation contributes to the progression of numerous chronic diseases, and plants are a rich source of bioactive secondary metabolites. In this study, bioassay-guided isolation of the previously unexplored Australian native plant *Beyeria viscosa* (Labill.) Miq. yielded eleven known compounds (**1**–**11**). The chemical structures of these compounds were identified by detailed spectroscopic data analysis, and definitive structural confirmation was established using single-crystal X-ray crystallography for fritillebic acid (**8**) and herbacetin 3,7,8-trimethyl ether (**5**) for the first time. All compounds were first screened for nitric oxide (NO) inhibitory activity and cytotoxicity in lipopolysaccharide (LPS) and interferon (IFN)-γ-stimulated RAW 264.7 macrophages, and the active NO inhibitors were further assessed for tumor necrosis factor-α (TNF-α) and interleukin-6 (IL-6) inhibition. Notably, compounds **5**, **8**, and **10** were evaluated for NO inhibitory activity for the first time, with compound **8** being the most potent (IC_50_ = 8.8 ± 1.3 μM), compound **10** showing moderate potency (IC_50_ = 12.2 ± 8.8 μM), and compound **5** being inactive. Among all tested compounds, fritillebic acid (**8**) emerged as the most active constituent, showing strong NO inhibition and moderate suppression of TNF-α and IL-6 production; therefore, it was further assessed in LPS-stimulated N-11 microglial cells, where it retained NO inhibitory activity (IC_50_ = 12.3 ± 0.5 μM) with a favorable activity–cytotoxicity profile (LC_50_ = 107.9 ± 1.9 μM). Consistent with the promising activity, molecular docking of compound **8** showed strong receptor-binding affinity with selected inflammation-related targets. Moreover, preliminary structure–activity relationship analysis of all isolated compounds suggested that substitution and oxygenation patterns may influence NO inhibitory potency. Overall, these findings identify fritillebic acid as the major anti-inflammatory lead from *B. viscosa* and highlight Australian native plants as a source of bioactive secondary metabolites.

## 1. Introduction

Inflammation is a fundamental defense mechanism triggered by the body in response to harmful stimuli such as pathogens, physical injury, or cellular damage [1]. Certain receptors in macrophages and microglial cells are activated in response to these stimuli, which release pro-inflammatory mediators, such as TNF-α, IL-6, and NO [2]. Although NO plays a beneficial role in host defense and immune regulation, its overproduction can become cytotoxic, contributing to oxidative stress, tissue damage, and the progression of chronic inflammation [3,4]. Therefore, inhibiting the overproduction of NO, TNF-α and IL-6 is considered a promising strategy in controlling inflammatory diseases.

Currently, the treatment of inflammatory disorders primarily relies on non-steroidal anti-inflammatory drugs (NSAIDs). However, the prolonged use of NSAIDs is often associated with significant gastrointestinal complications, including stomach ulcers and gastrointestinal bleeding. Moreover, certain NSAIDs, especially COX-2 inhibitors, have been linked to adverse cardiovascular outcomes such as elevated blood pressure, heart failure, thrombosis, and myocardial infarction [5,6]. Therefore, discovering new anti-inflammatory agents with improved safety profiles and fewer side effects remains a critical goal in the development of safer therapeutic alternatives.

Traditional medicines and plant-derived natural products present a promising alternative to conventional drugs, which are often associated with adverse side effects. These sources are considered valuable for identifying bioactive compounds that could serve as lead candidates in the development of therapies for inflammatory conditions [7,8]. Indigenous Australian communities have a rich history of using native plants for medicinal purposes, including sore throats, coughs, wounds, and skin infections [9,10]. Using ethnopharmacological knowledge documented by Dr. Frances Bodkin (*Dharawal* Elder), and through our ongoing screening program of Australian native plant extracts for anti-inflammatory activity we found *Beyeria viscosa* to be among one of the most potent candidates requiring further evaluation. This plant is commonly known as pinkwood or sticky wallaby bush, is an evergreen small tree that grows to a height of 6 m, belongs to the family Euphorbiaceae, and the entire genus is endemic to Australia. Although diterpenes, triterpenes, and flavonoids have been reported from other members of this genus, the chemical constituents of *B. viscosa* itself have not yet been investigated [11].

Herein, we report the isolation, structural identification and characterization of compounds (**1**–**11**) from the mature leaves of *B. viscosa* ethanolic extract using high-resolution mass spectrometry (HR-MS), nuclear magnetic resonance (NMR) spectroscopy, and X-ray crystallography. We further evaluate the anti-inflammatory activities of the isolated compounds by measuring their inhibitory effects on NO, TNF-α and IL-6 in IFN-γ and LPS stimulated RAW 264.7 macrophages and N-11 microglial cells. In addition, molecular docking study was performed to investigate the potential interactions of the most active compound with inflammatory target proteins, providing additional insight into its possible mechanism of action and therapeutic potential in inflammation and immune-related disorders. Preliminary structure–activity relationships of all isolated compounds were also explored to better understand the features associated with biological activity.

## 2. Materials and Methods

### 2.1. General Experimental Procedures

NMR spectra were recorded on a Bruker Avance 600 MHz spectrometer (Bruker Biospin GmbH, Rheinstetten, Germany), in the solvents indicated and referenced to residual ^1^H signals in deuterated solvents. HRMS was conducted using a Waters SYNAPT G2-Si mass spectrometer (Waters Corporation, Milford, MA, USA) operating in the positive and negative mode. An Agilent 1260 Infinity II coupled to a DAD detector (Agilent Technologies, Santa Clara, CA, USA) was used for HPLC analysis and compound separation.

### 2.2. Plant Material

Mature leaves of *B. viscosa* were collected from the Australian Botanic Garden, Mount Annan, NSW, Australia (34.0698° S, 150.7672° E) in June 2023, and a voucher specimen (A1999-0386) was deposited at the same place.

### 2.3. Extraction and Bioassay-Guided Isolation

The leaves of *B. viscosa* (598 g) were crushed using a hand blender and extracted with EtOH thrice until the solvent became colorless to afford an ethanolic extract (39.7 g). The extract was resuspended in EtOH and was subsequently subjected to an optimized semipreparative reversed-phase HPLC (Agilent 1260 Infinity II series) (Agilent Technologies, Santa Clara, CA, USA) on an Eclipse XDB-C8 [9.4 × 250 mm, 5 μm] column eluting at 2 mL/min with a gradient of 10−100% MeOH/H_2_O (modified with 0.01% formic acid) over 30 min with a hold at 100% MeOH for an additional 15 min and then equilibrated back to 10% MeOH/H_2_O in 1 min and held at this isocratic phase for an additional 6 min, to yield 19 fractions (Fr. 1−19). Each of these fractions was evaluated for its potential anti-inflammatory activity in LPS plus IFN-γ stimulated RAW 264.7 macrophages. From the 19 fractions, 4 were identified as pure fractions which included Fr. 6 (**1**), Fr. 9 (**2**), Fr. 11 (**5**), and Fr. 12 (**6**), corresponding to the following compounds: **1** (11.6 mg, *t*_R_ 19.9 min), **2** (13.1 mg, *t*_R_ 26.0 min), **5** (6.8 mg, *t*_R_ 28.8 min), and **6** (6.9 mg, *t*_R_ 29.9 min). In addition to this, Fr. 10 (18.6 mg, *t*_R_ 27.0 min) was further repurified on an Eclipse XDB-C8 [9.4 × 250 mm, 5 μm] column with an isocratic system of 58% MeOH/H_2_O (modified with 0.01% formic acid) over 30 min at a flow rate of 2 mL/min to afford two compounds, **3** (4.3 mg, *t*_R_ 15.1 min) and **4** (5.3 mg, *t*_R_ 15.9 min). Fr. 13 (80.2 mg, *t*_R_ 35.2 min) was also repurified using an Eclipse XDB-C8 [9.4 × 250 mm, 5 μm] column with a gradient elution of 10−100% MeOH/H_2_O (modified with 0.01% formic acid) at a flow rate of 2 mL/min over 20 min to yield compounds **7** (28.1 mg, *t*_R_ 9.2 min) and **8** (31.2 mg, *t*_R_ 13.9 min). Similarly, Fr. 14 (24.2 mg, *t*_R_ 35.1 min) was further purified using an Eclipse XDB-C8 [9.4 × 250 mm, 5 μm] column with a gradient system of 80–100% MeOH/H_2_O (modified with 0.01% formic acid) over 10 min and held at 100% MeOH for an additional 8 min, then equilibrated back to 80% MeOH within 1 min and maintained at 80% MeOH for an additional 5 min at a flow rate of 2.0 mL/min, affording compounds **9** (4.6 mg, *t*_R_ 11.2 min) and **10** (5.8 mg, *t*_R_ 9.9 min). Finally, compound **11** (11.9 mg, *t*_R_ 15.9 min) was acquired from Fr. 19 (25.2 mg, *t*_R_ 42.5 min) using an Eclipse XDB-C8 [9.4 × 250 mm, 5 μm] column, eluting with 100% MeOH over 25 min at a flow rate of 1 mL/min. Compounds **1** to **6** were isolated as the major constituents of the EtOH extract, with approximate isolated yields of 2–5% each, calculated based on the dry extract mass. Compounds **7**–**8,** and **10**–**11** were obtained in moderate yields (1–2%), whereas compound **9** was isolated only in trace amounts.

Kaempferol-3-*O*-rhamnoside (**1**): Yellow oil; UV (MeOH) λ_max_ 262, 343 nm; ^1^H NMR (600 MHz, DMSO-*d*_6_) δ 7.95 ppm (d, [*J* = 8.9 Hz], 2H, H-2′,6′), δ 6.92 ppm (d, [*J* = 8.9 Hz], 2H, H-3′,5′), δ 6.41 ppm (s, 1H, H-8), δ 6.20 ppm (s, 1H, H-6), δ 5.28 ppm (s, 1H, H-1″), δ 3.08–3.97 ppm (m, 4H, H2″-5″), and δ 0.78 ppm (d, [*J = 6.0* Hz], 3H, H_3_-6″); HRESI (+) MS ion *m*/*z* 455.0964 [M + Na]^+^, (calcd for C_21_H_20_O_10_Na; 455.0954).

Apigenin (**2**): White powder; UV (MeOH) λ_max_ 267, 335 nm; ^1^H NMR (600 MHz, DMSO-*d*_6_) δ 7.91 ppm (d, [*J* = 8.9 Hz], 2H, H-2′,6′), δ 6.91 ppm (d, [*J* = 8.9 Hz], 2H, H-3′,5′), δ 6.75 ppm (s, 1H, H-3), δ 6.45 ppm (s, 1H, H-8), δ 6.16 ppm (s, 1H, H-6), and δ 12.95 ppm (s, 1H, OH-5); HRESI (+) MS ion *m*/*z* 293.0426 [M + Na]^+^, (calcd for C_15_H_10_O_5_Na; 293.0426).

Demethoxysudachitin (**3**): Yellow crystalline powder; UV (MeOH) λ_max_ 278, 335 nm; ^1^H NMR (600 MHz, MeOD-*d*_4_) δ 8.04 ppm (d, [*J* = 8.9 Hz], 2H, H-2′,6′), δ 6.94 ppm (d, [*J* = 8.9 Hz], 2H, H-3′,5′), δ 6.25 ppm (s, 1H, H-3), δ 3.89 ppm (s, 3H, OMe-8)and δ 3.76 ppm (s, 3H, OMe-6); HRESI (+) MS ion *m*/*z* 331.0811 [M + H] ^+^, (calcd for C_17_H_15_O_7_; 331.0818).

Hispidulin (**4**): White solid; UV (MeOH) λ_max_ 262, 343 nm; ^1^H NMR (600 MHz, MeOD-*d*_4_) δ 7.98 ppm (d, [*J* = 8.9 Hz], 2H, H-2′,6′), δ 6.92 ppm (d, [*J* = 8.9 Hz], 2H, H-3′,5′), δ 6.40 ppm (s, 1H, H-3), δ 6.19 ppm (d, [*J* = 1.7 Hz], 1H, H-8), and δ 3.77 ppm (s, 3H, OMe-6); HRESI (+) MS ion *m*/*z* 323.0530 [M + Na]^+^, (calcd for C_16_H_12_O_6_Na; 323.0532).

Herbacetin 3,7,8-trimethyl ether (**5**): Yellow crystals; UV (MeOH) λ_max_ 253, 354 nm; ^1^H NMR (600 MHz, DMSO-*d*_6_) δ 7.95 ppm (d, [*J* = 8.9 Hz], 2H, H-2′,6′), δ 6.92 ppm (d, [*J =* 8.9 Hz], 2H, H-3′,5′), δ 6.57 ppm (s, 1H, H-6), δ 3.79 ppm (s, 3H, OMe-3), δ 3.80 ppm (s, 3H, OMe-8), and δ 3.91 ppm (s, 3H, OMe-7); HRESI (+) MS ion *m*/*z* 345.0979 [M + H] ^+^, (calcd for C_18_H_17_O_7_; 345.0974).

Genkwanin (**6**): Yellow crystalline powder; UV (MeOH) λ_max_ 254, 354 nm; ^1^H NMR (600 MHz, DMSO-*d*_6_) δ 7.95 ppm (d, [*J = 8.9* Hz], 2H, H-2′,6′), δ 6.93 ppm (d, [*J =* 8.9 Hz], 2H, H-3′,5′), δ 6.84 ppm (s, 1H, H-3), δ 6.77 ppm (d, [*J =* 2.1 Hz], 1H, H-8), δ 6.37 ppm (d, [*J* = 2.1 Hz], 1H, H-6), δ 3.86 ppm (s, 3H, OMe-7), and δ 12.97 ppm (s, 1H, OH-5); HRESI (+) MS ion *m/z* 285.0767 [M + H] ^+^, (calcd for C_16_H_13_O_5_; 285.0763).

Siegeskaurolic acid (**7**): White powder; UV (MeOH) λ_max_ 235 nm; ^1^H NMR (600 MHz, DMSO-*d*_6_) δ 12.09 ppm (s, 1H, 19-COOH), δ 3.12 ppm (d, [*J* = 7.5 Hz], 2H, H_2_-17), δ 2.35 ppm (br s, 1H, H-13), δ 1.98 ppm (m, 1H, H-3a), δ 1.77 ppm (m, 4H, overlapped, H-1a, H-3b, H-6b, H-16), δ 1.72 ppm (m, 1H, H-2b), δ 1.69 ppm (m, 2H, H_2_-14), δ 1.53 ppm (m, 1H, H-11a), δ 1.44 ppm (m, 1H, H-11b), δ 1.43 ppm (m, 2H, H_2_-12), δ 1.42 ppm (m, 1H, H-15a), δ 1.41 ppm (m, 1H, H-7b), δ 1.36 ppm (m, 1H, H-7a), δ 1.34 ppm (m, 1H, H-6a), δ 1.32 ppm (m, 1H, H-2a), δ 1.08 ppm (s, 3H, H_3_-19), δ 0.99 ppm (m, 1H, H-5), δ 0.94 ppm (m, 1H, H-9), δ 0.85 ppm (s, 3H, H_3_-20), δ 0.80 ppm (m, 1H, H-15b), and δ 0.76 ppm (m, 1H, H-1b); HRESI (+) MS ion *m*/*z* 343.2244 [M + Na]^+^, (calcd for C_20_H_32_O_3_Na; 343.2249).

Fritillebic acid (**8**): Colorless crystals; UV (MeOH) λ_max_ 229 nm; ^1^H NMR (600 MHz, DMSO-*d*_6_) δ 11.93 ppm (br s, 1H, 17-COOH), δ 4.35 ppm (dd, [*J* = 4.8, 4.8 Hz], 1H, H-3), δ 2.50 ppm (br s, 1H, H-16), δ 2.37 ppm (br s, 1H, H-13), δ 1.99 ppm (s, 3H, 3-OC(O)CH_3_), δ 1.80 ppm (d, [*J* = 10.5 Hz], 1H, H-1a), δ 1.62 ppm (m, 1H, H-2a), δ 1.58 ppm (m, 2H, H_2_-15), δ 1.54 ppm (m, 2H, overlapped, H-2b, H-11a), δ 1.50 ppm (m, 3H, overlapped, H-7a, H-11b, H-12a), δ 1.49 ppm (m, 1H, H-6a), δ 1.45 ppm (m, 1H, H-12b), δ 1.44 ppm (m, 1H, H-7b), δ 1.32 ppm (m, 1H, H-6b), δ 1.04 ppm (dd, [*J* = 5.5, 5.5 Hz], 1H, H-14a), δ 0.99 ppm (d, [*J* = 5.8 Hz], 1H, H-9), δ 0.97 ppm (s, 3H, H_3_-20), δ 0.89 ppm (d, [*J* = 4.5 Hz], 1H, H-14b), δ 0.87 ppm (s, 1H, H-1b), δ 0.83 ppm (d, [*J* = 11.5 Hz], 1H, H-5), and δ 0.80 ppm (s, 6H, H_3_-18, H_3_-19); HRESI (+) MS ion *m*/*z* 363.2548 [M + H] ^+^, (calcd for C_22_H_35_O_4_; 363.2535).

Kaurenoic acid (**9**): Greenish solid; UV (MeOH) λ_max_ 215 nm; ^1^H NMR (600 MHz, MeOD-*d*_4_) δ 4.78 ppm (s, 1H, H-17a), δ 4.72 ppm (s, 1H, H-17b), δ 2.12 ppm (m, 1H, H-3b), δ 1.98 ppm (br s, 2H, H_2_-15), δ 1.65 ppm (m, 1H, H-14b), δ 1.59 ppm (m, 1H, H-2b), δ 1.56 ppm (m, 1H, H-1b), δ 1.51 ppm (m, 2H, H_2_-6), δ 1.47 ppm (m, 1H, H-12b), δ 1.43 ppm (m, 2H, H_2_-11), δ 1.40 ppm (m, 2H, H_2_-7), δ 1.38 ppm (m, 1H, H-12a), δ 1.34 ppm (m, 1H, H-2a), δ 1.17 ppm (s, 3H, H_3_-18), δ 1.09 ppm (m, 1H, H-14a), δ 1.06 ppm (m, 1H, H-5), δ 0.98 ppm (s, 3H, H_3_-20), δ 0.96 ppm (m, 1H, H-9), δ 0.94 ppm (m, 1H, H-3a), and δ 0.83 ppm (ddd, [*J* = 13.0, 12.9, 5.5 Hz], 1H, H-1a); HRESI (+) MS ion *m/z* 325.2148 [M + Na] ^+^, (calcd for C_20_H_30_O_2_Na; 325.2143).

Lup-20(29)-ene-3β,16β-diol (**10**): White needle crystals; UV (MeOH) λ_max_ 229 nm; ^1^H NMR (600 MHz, MeOD-*d_4_*) data δ 4.70 ppm (s, 1H, H-29a), δ 4.58 ppm (s, 1H, H-29b), δ 3.54 ppm (dd, [*J* = 4.8, 4.8 Hz], 1H, H-16), δ 3.12 ppm (dd, [*J* = 4.8, 4.8 Hz], 1H, H-3), δ 2.51 ppm (dt, [*J* = 5.8, 11.2 Hz], 1H, H-19), δ 1.96 ppm (m, 1H, H-21a), δ 1.69 (s, 4H, overlapped, H-13 and H_3_-30), δ 1.68 ppm (m, 1H, H-22a), δ 1.60 ppm (m, 2H, overlapped, H-1a, H-11a), δ 1.58 ppm (m, 1H, H-1b), δ 1.56 ppm (m, 2H, H_2_-2), δ 1.49 ppm (m, 1H, H-7b), δ 1.47 ppm (m, 1H, H-6a), δ 1.44 ppm (m, 1H, H-6b), δ 1.40 ppm (m, 1H, H-7a), δ 1.38 ppm (m, 1H, H-18), δ 1.30 ppm (m, 6H, overlapped, H-9, H-11b, H_2_-12, H-21b, H-22b), δ 1.23 ppm (d, [*J =* 9.9 Hz], 1H, H-15a), δ 1.07 ppm (s, 3H, H_3_-26), δ 1.02 ppm (s, 3H, H_3_-27), δ 0.95 ppm (s, 3H, H_3_-24), δ 0.87 ppm (s, 3H, H_3_-25), δ 0.79 ppm (s, 3H, H_3_-28), δ 0.76 ppm (s, 3H, H_3_-23), and δ 0.71 ppm (d, [*J* = 9.9 Hz], 1H, H-5); HRESI (+) MS ion *m*/*z* 443.3889 [M + H] ^+^, (calcd for C_30_H_51_O_2_; 443.3889).

Lupeol (**11**): White powder; UV (MeOH) λ_max_ 230 nm; ^1^H NMR (600 MHz, CDCl_3_) data δ 4.69 ppm (s, 1H, H-29a), δ 4.45 ppm (s, 1H, H-29b), δ 3.19 ppm (dd, [*J* = 4.9, 4.9 Hz], 1H, H-3), δ 2.38 ppm (dt, [*J* = 5.8, 11.1 Hz], 1H, H-19), δ 1.92 ppm (m, 1H, H-21a), δ 1.68 ppm (br s, 3H, H_3_-30), δ 1.67 ppm (m, 1H, H-15a), δ 1.66 ppm (m, 1H, H-13), δ 1.65 ppm (m, 1H, H-22a), δ 1.56 ppm (m, 2H, H_2_-2), δ 1.52 ppm (m, 1H, H-6a), δ 1.49 ppm (m, 1H, H-7a), δ 1.48 ppm (m, 1H, H-1a), δ 1.41 ppm (m, 2H, H_2_-11), δ 1.39 ppm (m, 3H, overlapped, H-6b, H_2_-16), δ 1.34 ppm (m, 3H, overlapped, H-1b, H-7b, H-18), δ 1.26 ppm (m, 2H, overlapped, H-9, H-21b), δ 1.21 ppm (m, 2H, H_2_-12), δ 1.06 ppm (m, 1H, H-15b), δ 1.03 ppm (s, 3H, H_3_-27), δ 0.97 ppm (s, 3H, H_3_-23), δ 0.94 ppm (s, 3H, H_3_-26), δ 0.89 ppm (m, 1H, H-22b), δ 0.87 ppm (s, 3H, H_3_-25), δ 0.79 ppm (s, 3H, H_3_-28), δ 0.76 ppm (s, 3H, H_3_-24), and δ 0.68 ppm (d, [*J* = 9.9 Hz], 1H, H-5); HRESI (+) MS ion *m/z* 427.3941 [M + H] ^+^, (calcd for C_30_H_51_O; 427.3940).

### 2.4. X-Ray Crystallographic Analysis

Crystals of compounds **5** and **8** were obtained using a combination of slow cooling, slow evaporation, and vapor diffusion techniques, following standard procedures for organic molecule crystallization [12,13]. The single crystal data for both compounds were collected at the Australian Synchrotron on the MX1 beamline using silicon double crystal monochromated radiation (λ = 0.71073 Å) at 293 K [14]. The XDS software package [15] was used on site for data integration, processing, and scaling. An empirical absorption correction was applied with SADABS (version 2014/5; Bruker AXS Inc.) [16]. The structures were solved by intrinsic phasing, and refinements were carried out using a suite of SHELX programs [17,18], via the Olex2 graphical interface [19]. Crystallographic data of **5** (CCDC number: 2529172) and **8** (CCDC number: 2529171) were deposited at the Cambridge Crystallographic Data Centre. Additional crystallographic information is available in the Supporting Materials (Tables S1–S4).

Crystal Data for **5** C_18_H_16_O_7_ (M = 344.31 g/mol); monoclinic, 0.02 × 0.01 × 0.01 mm^3^, space group *P2*_1_/*n*, *V* = 1512.1(5) Å^3^, Z = 4, D_c_ = 1.512 g/cm^3^, *F*(000) = 720.0, Mo Kα radiation, λ = 0.71073 Å, *T* = 293(2) K, µ = 0.118 mm^−1^; 2θ _range_ = 2.97 to 57.298, 27505° reflections collected, 3373 unique (*R*_int_ = 0.0455); final GooF = 1.029, R1 = 0.0539 [*I* > 2σ (I)], wR2 = 0.1568.

Crystal Data for **8** C_22_H_33_O_4_ (M = 361.48 g/mol); monoclinic, 0.02 × 0.01 × 0.01 mm^3^, space group *C*2, *V* = 1989.8(7) Å^3^, Z = 4, D_c_ = 1.207 g/cm^3^, *F*(000) = 788.0, Mo Kα radiation, λ = 0.71073 Å, *T* = 293(2) K, µ = 0.081 mm^−1^; 2θ _range_ = 1.68 to 57.39, 17732° reflections collected, 4234 unique (*R*_int_ = 0.0819); final GooF = 1.037, R1 = 0.1053 [*I* > 2σ (I)], wR2 = 0.2264.

### 2.5. Maintenance of RAW 264.7 Macrophages and N-11 Microglial Cells

Both cell lines were cultured separately in 75 cm^2^ flasks in Dulbecco’s Modified Eagle Medium (DMEM; Gibco, Thermo Fisher Scientific, Waltham, MA, USA) supplemented with 10% fetal bovine serum (FBS; Life Technologies, Carlsbad, CA, USA), L-glutamine (2 mM; Sigma Life Science, Poole, UK), and penicillin–streptomycin (100 μg/mL; Life Technologies, Carlsbad, CA, USA), and maintained in 5% CO_2_ at 37 °C, with media being replaced every 3–4 days. Upon reaching confluence, RAW 264.7 cells were subcultured by gentle scraping using a rubber policeman, whereas N-11 cells were detached using trypsin-EDTA (Life Technologies, Carlsbad, CA, USA), then centrifuged and resuspended in the culture medium.

### 2.6. Pro-Inflammatory Activation of Cells

RAW 264.7 cells (1 × 10^6^ cells/mL) were seeded in 96-well plates (Corning Costar, Sigma, Sydney, Australia) with 100 μL per well and incubated overnight or until reaching confluency. Once the cells reached confluency, each compound was serially diluted from a starting concentration of 100 μg/mL to generate a dose response curve (i.e., 100, 50, 25, 12.5, 6.25, and 3.13 μg/mL), and co-incubated with the cells for 1 h prior to stimulation with lipopolysaccharide (LPS; Sigma-Aldrich, Life Sciences, St. Louis, MO, USA) and interferon-γ (IFN-γ; PeproTech, Brisbane, Australia), each at a final concentration of 50 ng/mL. N-11 microglial cells were treated under similar conditions, except that cells were stimulated with LPS only at a final concentration of 1 µg/mL. Following stimulation, both cell lines were incubated for 24 h at 37 °C with 5% CO_2_. The supernatant was then collected for NO, TNF-α and IL-6 assays, and cell viability was assessed using the Alamar Blue assay. Nonactivated cells (exposed to media only) were used as the negative control, and activated cells treated with curcumin (concentration range of 8.48−277.6 μM) were used as the positive control.

### 2.7. Determination of Nitrite by the Griess Assay

NO was determined by the Griess reagent as described in previous studies [20]. Griess reagent was freshly made up of equal volumes of 0.1% *N*-1-naphthylethylenediamine dihydrochloride in Milli-Q water (Merck Millipore, Burlington, MA, USA) and 1% sulfanilamide in 5% phosphoric acid. Following drug treatment and stimulation with LPS and IFN-γ, 100 μL of supernatant was transferred to a fresh 96-well plate and mixed with 100 μL of Griess reagent. The production of nitrite as an indicator of NO was measured at 540 nm in a POLARstar Omega microplate reader (BMG Labtech, Mornington, Australia).

### 2.8. Determination of TNF-α by ELISA

The stored supernatants were used for determination of TNF-α using a sandwich ELISA according to manufacturer’s instructions as described previously (PeproTech Asia, Brisbane, Australia) with slight modifications [21]. The capture antibody was used at a concentration of 50 μg/mL in Milli-Q water. Serial dilutions of TNF-α standard (0–10,000 pg/mL) were prepared in diluent buffer (0.05% Tween-20, 0.1% BSA in PBS) and used to generate a standard curve. TNF-α levels in the samples were detected using a biotinylated secondary antibody, followed by an avidin–peroxidase conjugate and 3,3′,5,5′-tetramethylbenzidine (TMB) as the chromogenic substrate. Color development was monitored at 655 nm with readings taken every 5 min. After approximately 20 min, the reaction was terminated by adding 0.5 M sulfuric acid, and absorbance was measured at 455 nm using a POLARstar Omega microplate reader (BMG Labtech, Mornington, Australia). TNF-α concentrations were calculated from the standard curve, generated using nonlinear regression analysis in GraphPad Prism.

### 2.9. Determination of IL-6 by ELISA

The stored supernatants were analyzed for IL-6 determination using a commercial ELISA kit (PeproTech, Queensland, Australia) according to the manufacturer’s instructions, as previously described [22]. The standard murine IL-6 (0–4000 pg/mL) from the kit was used for calibration curve. The absorbance was measured at 410 nm and concentrations of IL-6 in the experimental samples were extrapolated from a standard curve.

### 2.10. Determination of Cell Viability by the Alamar Blue Assay

A 100 μL portion of Alamar Blue solution [10% Alamar Blue (0.1 mg/mL resazurin) in DMEM media] was added to cells 24 h after the LPS and IFN-γ stimulation and incubated at 37 °C for 2 h [23]. The fluorescence intensity was measured with excitation at 530 nm and emission at 590 nm using a microplate reader. The results were expressed as a percentage of the intensity to that of control cells (nonactivated cells).

### 2.11. Molecular Docking Study

The 3D structure of fritillebic acid (**8**) was retrieved from the PubChem database and imported into Chem3D for energy minimization. The optimized ligand structure was saved in PDB format. The 3D crystal structures of the target proteins were downloaded from the Protein Data Bank (PDB IDs: 5IJC, 4KIK, and 4NOS, respectively). Suitable protein structures were selected based on resolution, structural completeness, and the presence of co-crystallized ligand information. Protein structures were prepared and analyzed using Discovery Studio (2015), PyMOL 3.1.8, and MGLTools 1.5.7. Water molecules and original ligand were removed, hydrogen atoms were added, charges were calculated, and the processed ligands and receptor files were converted into PDBQT format for docking analysis. All rotatable bonds in the ligand were defined as flexible, while the receptors were kept rigid. Docking simulations were carried out using AutoDock Vina 1.1.2 implemented in MGLTools 1.5.7. The docking poses, binding sites, and interacting amino acid residues were subsequently visualized and analyzed using PyMOL 3.1.8.

### 2.12. Statistical Analysis

Data analysis was carried out using GraphPad Prism 10.5.0. Calculations were performed using Microsoft Excel version 16.61.1. IC_50_ values were obtained by using the sigmoidal dose response function in GraphPad Prism. The results were expressed as mean ± standard deviation (SD).

## 3. Results and Discussion

### 3.1. Bioassay-Guided Isolation

In our previous study, *B. viscosa’s* ethanolic extract exhibited potent anti-inflammatory activity, which led us to do its chemical profiling by bioassay-guided isolation approach. Firstly, the ethanolic extract (39.7 g) was fractioned by semipreparative reversed-phase HPLC (Agilent 1260 Infinity II series) on an Eclipse XDB-C8 [9.4 × 250 mm, 5 μm] column, eluting at 2 mL/min with a gradient of 10−100% MeOH/H_2_O (modified with 0.01% formic acid) over 30 min with a hold at 100% MeOH for an additional 15 min and then equilibrated back to 10% MeOH/H_2_O in 1 min and held at this isocratic phase for an additional 6 min, to yield 19 fractions (Figure S23). After the anti-inflammatory and cytotoxicity screening (Table S5), bioactive fractions were subsequently subjected to further purification under the conditions detailed in the methodology section (extraction and bioactivity-guided isolation), resulting in the isolation of 11 known compounds (**1**–**11**).

### 3.2. Identification and Characterization

Following the bioactivity-guided isolation, the structures of compounds were identified by the analysis of 2D NMR, HRESI-MS (+), and a close comparison with published data (see Supplementary Materials, Figures S1–S22). These compounds were identified as kaempferol-3-*O*-rhamnoside (**1**) [24,25], apigenin (**2**) [26], demethoxysudachitin (**3**) [27], hispidulin (**4**) [28,29], herbacetin 3,7,8-trimethyl ether (**5**) [30,31], genkwanin (**6**) [32,33], siegeskaurolic acid (**7**) [34], fritillebic acid (**8**) [35], kaurenoic acid (**9**) [36], lup-20(29)-ene-3β,16β-diol (**10**) [37,38], and lupeol (**11**) [39,40]. Structural representation of these compounds is shown in Figure 1.

**Figure 2:**
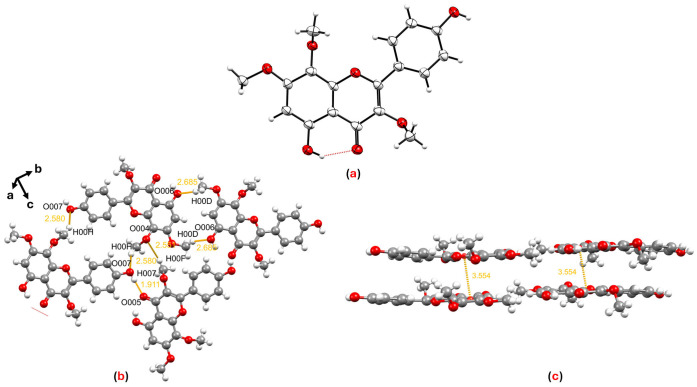
(**a**) ORTEP diagram of herbacetin 3,7,8-trimethyl ether (**5**); (**b**) crystal packing of compound **5** showing hydrogen-bonding interactions; and (**c**) crystal packing of compound **5** illustrating π···π stacking interactions.

After identifying the planar structures, we successfully characterized the structures of compounds **5** and **8** by single-crystal X-ray diffraction (SCXRD) for the first time (Tables S1–S4). For herbacetin 3,7,8-trimethyl ether (**5**), SCXRD conclusively established the precise regio-chemistry of its three methoxy substituents (Figure 2a). The crystal lattice of this compound is constituted by a series of hydrogen-bonding interactions occurring along the crystallographic *bc* plane between O007···H00H, H007···O005, O004···H00F and H00D···O006 at distances of 2.580, 1.911, 2.551 and 2.685 Å, respectively (Figure 2b). In addition, π···π stacking interactions are observed between adjacent pyran and benzene rings along the *a*-axis, with a centroid–centroid distance of 3.554 Å (Figure 2c). While this compound has been previously isolated from *Larrea tridentata*, its structure was assigned based solely on spectroscopic evidence [31]. Our crystallographic data provide definitive structural confirmation and a reliable reference for future studies.

**Figure 3:**
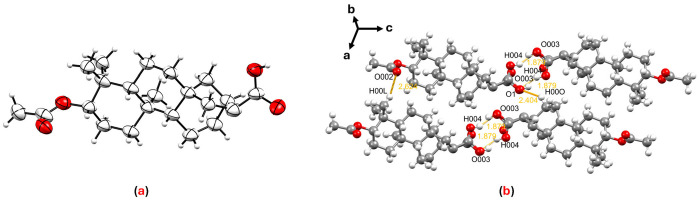
(**a**) ORTEP diagram of fritillebic acid (**8**); and (**b**) crystal packing of compound **8** showing hydrogen-bonding interactions.

Similarly, SCXRD analysis of fritillebic acid (**8**) provided unambiguous structural confirmation (Figure 3a). The crystal lattice of this compound is stabilized by intermolecular hydrogen-bonding interactions occurring along the crystallographic *ac* plane between H004···O003 at a distance of 1.879 Å. Additional hydrogen-bonding interactions are observed along the *ab* plane between O002···H00L at a distance of 2.624 Å and along the *bc* plane between O1···H00O at a distance of 2.404 Å (Figure 3b). Although this compound was previously reported from *Fritillaria ebeiensis* and characterized by spectroscopic methods [35], the present study constitutes the first single-crystal X-ray diffraction report of fritillebic acid (**8**). This three-dimensional structural information provides a valuable foundation for future structure–activity relationship studies.

### 3.3. Anti-Inflammatory Activity

The cytotoxicity of compounds **1** – **11** was first evaluated in RAW 264.7 macrophages with various treatments, and our results showed that the LC_50_ values were all above 28.9 ± 5.1 μM. All compounds were then tested for anti-inflammatory activity by assessing their ability to inhibit NO production in RAW 264.7 macrophages activated with LPS and IFN-γ. As shown in Table 1, compound **8** exhibited strong NO inhibitory activity, with an IC_50_ value of 8.8 ± 1.3 μM, comparable to positive control curcumin (IC_50_ 8.5 ± 1.2 μM). Furthermore, compound **8** showed a higher therapeutic index (TI = 8.7) than curcumin (TI = 4.0), indicating a more favorable balance between NO inhibitory activity and cytotoxicity. Additionally, compounds **2**, **3**, **4**, **9** and **10** exhibited moderate NO inhibition, with IC_50_ values ranging from 12.2 to 25.6 μM, whereas the remaining compounds were inactive (IC_50_ > 100 μM). Based on NO inhibitory activity, active compounds (**2**, **3**, **4**, **8**, **9** and **10**) were further evaluated for TNF-α suppression. Among them, compounds **3** and **10** were inactive (IC_50_ > 100 μM), while the others showed moderate activity with IC_50_ values ranging from 15.2 to 43.8 μM (Table 1). The most active compound, fritillebic acid (**8**), was further assessed for its effect on IL-6 production in RAW 264.7 macrophage supernatants by ELISA, and inhibited IL-6 production with an IC_50_ value of 34.0 ± 5.5 μM. The moderate effects of compound **8** on TNF-α and IL-6 production indicate that it may have a greater influence on pathways associated with iNOS-mediated NO production than on broader cytokine release. Concentration response curves for NO inhibition, cytotoxicity, TNF-α, and IL-6 inhibition for corresponding compounds (**1**–**11**) are provided in the Supplementary Information (Figures S24 and S25).

Based on the promising anti-inflammatory activity of fritillebic acid (**8**) in RAW 264.7 macrophages, it was further evaluated for NO production and cell viability in LPS-stimulated N-11 microglial cells. In this model, compound **8** also showed potent NO inhibitory activity with an IC_50_ value of 12.3 ± 0.5 μM, and an LC_50_ value of 107.9 ± 1.9 μM in the cell viability. Curcumin was used as a reference compound for comparison and showed comparable NO inhibitory activity (IC_50_ = 11.7 ± 0.3 μM) but a lower LC_50_ value (57.0 ± 1.6 μM), indicating that compound **8** had a more favorable activity–cytotoxicity profile.

**Figure 4:**
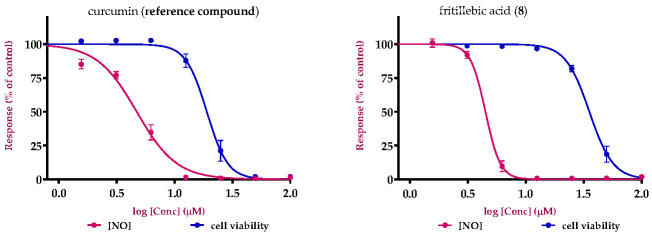
Concentration response curves of fritillebic acid (**8**) and curcumin (reference compound) in LPS-stimulated N-11 microglial cells showing inhibition of NO production (IC_50_) and cell viability (LC_50_). Data are presented as mean ± SD of 3 individual experiments in triplicate. IC_50_ and LC_50_ values were calculated by sigmoidal dose response function.

The evaluation of compound **8** in N-11 microglial cells further supports its relevance to neuroinflammatory processes. Microglia are the resident immune cells of the central nervous system and play a central role in neuroinflammation through the release of inflammatory mediators such as NO and pro-inflammatory cytokines. These findings suggest that the anti-inflammatory activity of compound **8** is not restricted to macrophages but extends to microglial cells, supporting its potential relevance in neuroinflammation-associated inflammatory responses. Concentration response curves for NO inhibition and cell viability for compound **8** and curcumin are shown in Figure 4.

To the best of our knowledge, compounds **5**, **8**, and **10** have not previously been evaluated for anti-inflammatory activity, and compounds **5** and **8**, in particular, have not been reported in any prior biological activity studies. Among these, fritillebic acid (**8**) exhibited the strongest anti-inflammatory activity in both RAW 264.7 macrophages and N-11 microglial cells, and thus represents a promising lead compound. Lup-20(29)-ene-3β,16β-diol, (**10**) exhibited moderate inhibitory activity, whereas herbacetin 3,7,8-trimethyl ether (**5**) was inactive but remains a suitable candidate for broader pharmacological profiling to define its bioactivity beyond NO/TNF-α endpoints.

In contrast, for compounds with previously reported anti-inflammatory activity, our results were broadly consistent with the literature, where apigenin (**2**), demethoxysudachitin (**3**), hispidulin (**4**), and kaurenoic acid (**9**) have been reported to produce measurable suppression of NO production in activated macrophages [41,42,43,44]. Kaempferol-3-O-rhamnoside (**1**) is commonly reported as inactive [45], and although genkwanin (**6**), siegeskaurolic acid (**7**), and lupeol (**11**) have shown anti-inflammatory effects in some macrophage assays [46,47,48], they were weak under our experimental conditions. Minor differences in potency relative to reported IC_50_ values are expected and likely reflect variations in assay parameters such as incubation time, LPS/IFN-γ concentration, cell density, solubility and cytotoxicity thresholds [49].

Overall, the identification of previously untested compounds with significant NO inhibitory activity expands the pharmacological knowledge of *Beyeria viscosa*. In particular, the activity of fritillebic acid (**8**) in both macrophage and microglial inflammatory models supports its potential relevance to inflammation and neuroinflammation related research. These findings strengthen the value of Australian native plants as reservoirs of structurally diverse bioactive compounds and provide a solid basis for future mechanistic and preclinical studies.

### 3.4. Molecular Docking Analysis

Molecular docking was performed to investigate the potential inflammatory targets associated with the activity of the most active compound, fritillebic acid (**8**). Compound **8** was docked against three inflammation-related target proteins, toll-like receptor 4–myeloid differentiation factor 2 (TLR4–MD-2), inhibitor of nuclear factor kappa-B kinase subunit beta (IKKβ), and inducible nitric oxide synthase (iNOS). The docking results showed favorable predicted binding affinities toward all three targets, with binding energies of −9.1, −7.3, and −6.4 kcal/mol for TLR4–MD-2, iNOS, and IKKβ, respectively (Table 2).

**Figure 5:**
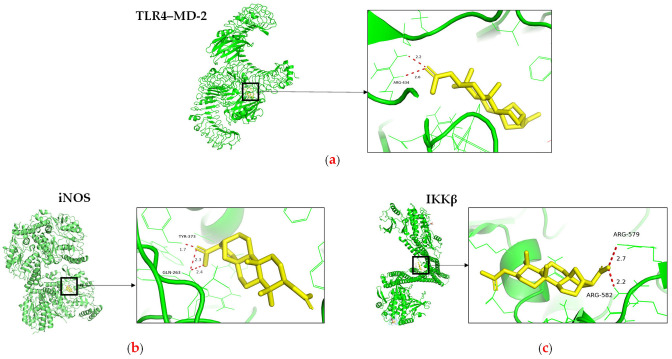
Molecular docking poses of fritillebic acid (**8**) with inflammation-related target proteins: (**a**) TLR4–MD-2, (**b**) iNOS, and (**c**) IKKβ. For each target, the whole-protein view shows the predicted binding location, and the enlarged view shows the binding-site interactions. Compound **8** is shown in yellow, protein structures are shown in green, and hydrogen-bonding interactions with key amino acid residues are indicated by red dashed lines.

The strongest predicted binding affinity was observed toward TLR4–MD-2, where compound **8** formed a hydrogen-bonding interaction with ARG-434 (Figure 5a), suggesting that it may interact with the LPS-recognition complex and potentially interfere with upstream inflammatory activation. Since LPS stimulation through TLR4–MD-2 activates downstream inflammatory signaling pathways, including NF-κB/MAPK signaling, this interaction may contribute to the overall anti-inflammatory response observed in the cellular assays. In addition, compound **8** showed favorable predicted binding affinity toward iNOS (−7.3 kcal/mol), with interactions involving the amino acid residues TYR-373 and GLN-263 (Figure 5b), which are located within or adjacent to the L-arginine substrate-binding region of iNOS. Since iNOS catalyzes the production of NO from L-arginine during inflammatory activation, the localization of compound 8 near this catalytic region is consistent with the strong NO inhibitory activity observed in the cellular assay. The interaction with TYR-373 may contribute to ligand stabilization within the catalytic cavity through hydrophobic or π-related interactions, while GLN-263 may support ligand positioning through hydrogen-bonding interactions. Together, these predicted interactions suggest that compound **8** may interfere with substrate access or stabilization within the iNOS catalytic pocket, thereby contributing to reduced NO production. The comparatively weaker predicted interaction with IKKβ (−6.4 kcal/mol), involving ARG-579 and ARG-582 (Figure 5c), may indicate partial modulation of NF-κB-related signaling, which could be associated with the moderate suppression of TNF-α and IL-6 observed in the cellular assays. Overall, these docking results provide supportive evidence that fritillebic acid may exert its anti-inflammatory activity through modulation of both upstream LPS-mediated signaling and downstream NO-producing pathways.

### 3.5. Structure–Activity Relationship (SAR)

For SAR analysis of NO inhibition, compounds were categorized into three classes, flavonoids (**1**–**6**), diterpenoids (**7** and **9**), and triterpenoids (**10** and **11**), and the results are summarized in Table 3. In the flavonoid series, NO-inhibitory activity was observed only when the C-3 position was unsubstituted and the C-7 hydroxyl group remained free. This trend is exemplified by apigenin (**2**), demethoxysudachitin (**3**), and hispidulin (**4**), which both possess a free 7-OH group and an unsubstituted C-3 position (3-H), and accordingly showed good activity (IC_50_ 16.4 ± 1.5 µM, 25.6 ± 3.2 µM and 17.6 ± 4.6 µM, respectively).

In contrast, masking these positions through glycosylation or methylation was associated with loss of activity (IC_50_ > 100 µM) in the remaining flavonoids (**1**, **5** and **6**), suggesting that these positions must remain unmasked to enable productive target binding or cellular engagement. This interpretation is consistent with prior reports showing that flavonoid glycosides are weak inhibitors and that substitution patterns strongly influence NO suppression [41,50].

For the kaurane diterpenoids **7** and **9**, modification at the C-17 terminus created a pronounced activity cliff. Despite sharing the same kaurane core and a conserved carboxylic acid function, kaurenoic acid (**9**) bearing an exocyclic C-17 methylene was active (IC_50_ 14.1 ± 0.3 µM), whereas siegeskaurolic acid (**7**) carrying a C-17 hydroxyl (17-OH) was inactive (IC_50_ > 100 µM). This contrast highlights C-17 as a key SAR hotspot, suggesting that C-17 unsaturation is favorable for NO inhibition, while C-17 hydroxylation is detrimental.

Finally, within the lupane triterpenoids, C-16 substitution emerged as a key potency determinant. Introduction of a 16β-hydroxyl group in lup-20(29)-ene-3β,16β-diol (**10**) increased activity (IC_50_ 12.2 ± 8.8 µM) compared with the corresponding non-hydroxylated analogue lupeol (**11**) (IC_50_ 92.8 ± 10.1 µM), representing an 8-fold improvement. This activity gain highlights C-16 oxygenation as a favorable SAR feature in the lupane scaffold, plausibly by increasing polarity and adding hydrogen-bonding capacity. This observation aligns with reports that functionalized lupeol-type triterpenoids suppress LPS-induced NO production in macrophages [38].

## 4. Conclusions

This study represents the first comprehensive phytochemical investigation of the Australian native plant *Beyeria viscosa*. Bioassay-guided isolation and structural identification of eleven compounds revealed that this species is a rich source of flavonoid and terpene metabolites. Anti-inflammatory evaluation of the isolated constituents further expanded the pharmacological knowledge of this plant, representing the first assessment of compounds **5**, **8**, and **10** for this activity.

Among the isolated compounds, fritillebic acid (**8**) emerged as the most active constituent, showing potent inhibition of NO production in RAW 264.7 macrophages and retaining activity in N-11 microglial cells. These findings support the potential relevance of compound **8** in both peripheral inflammatory and neuroinflammatory models. Molecular docking analysis provided additional supportive evidence by indicating possible interactions of compound **8** with key inflammation-related targets, including TLR4–MD-2, iNOS, and IKKβ.

In addition to the biological findings, SCXRD analysis confirmed the structures of herbacetin 3,7,8-trimethyl ether (**5**) and fritillebic acid (**8**) for the first time. The crystallographic confirmation of compound **8**, the most active compound in this study, provides an important structural basis for future mechanistic, pharmacological, and structure-based investigations.

Preliminary structure–activity relationship analysis of all isolated compounds suggested that substitution and oxygenation patterns may influence anti-inflammatory activity. Overall, this study highlights *B. viscosa* as a significant phytochemical and pharmacological potential. The identification of fritillebic acid (**8**) as a promising anti-inflammatory natural product lead supports further investigation of this compound and reinforces the importance of Australian native plants as valuable sources for the discovery of novel bioactive molecules.

## Figures and Tables

**Figure 1:**
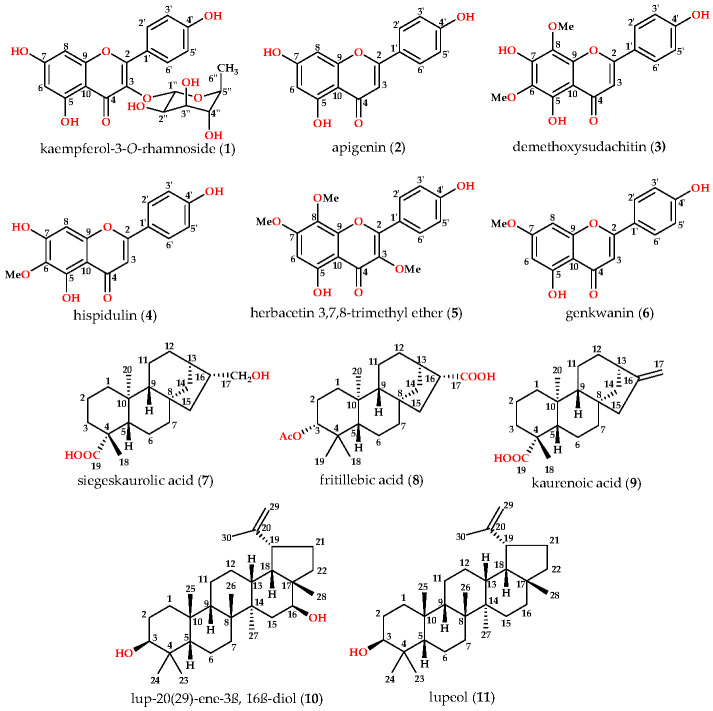
Chemical structures of compounds **1***–***11** from *B. viscosa.*

**Table 1:** In vitro anti-inflammatory activity of compounds (**1**–**11**) in LPS and IFN-γ-activated RAW 264.7 macrophages.

Compound	Inhibition of NO Production (IC_50_) (μM)	Inhibition of TNF-α Production (IC_50_) (μM)	Cytotoxicity (LC_50_) (μM)	Therapeutic Index (in Comparison to NO Inhibition)
curcumin (**+ve control**)	8.5 ± 1.2	10.1 ± 0.5	34.4 ± 5.1	4.0
kaempferol-3-*O*-rhamnoside (**1**)	>100	NT	>100	>1.0
apigenin (**2**)	16.4 ± 1.5	20.1 ± 3.8	62.1 ± 6.8	3.7
demethoxysudachitin (**3**)	25.6 ± 3.2	>100	>100	>3.9
hispidulin (**4**)	17.6 ± 4.6	43.8 ± 1.9	>100	>5.6
herbacetin 3,7,8-trimethyl ether (**5**)	>100	NT	>100	>1.0
genkwanin (**6**)	>100	NT	>100	>1.0
siegeskaurolic acid (**7**)	>100	NT	>100	>1.0
fritillebic acid (**8**)	8.8 ± 1.3	37.9 ± 3.5	76.6 ± 8.8	8.7
kaurenoic acid (**9**)	14.1 ± 0.3	15.2 ± 1.7	37.5 ± 3.3	2.6
lup-20(29)-ene-3β,16β-diol (**10**)	12.2 ± 8.8	>100	28.9 ± 5.1	2.0
lupeol (**11**)	92.8 ± 10.1	NT	>100	>1.0

“NT” means not tested; results represent mean ± SD of 3 individual experiments in triplicate.

**Table 2:** Molecular docking binding energies of fritillebic acid (**8**) against TLR4-MD-2, iNOS and IKKβ.

Target Protein	PDB ID	Binding Energy (kcal/mol)	Amino Acid Residues
TLR4-MD-2	5IJC	−9.1	ARG-434
iNOS	4NOS	−7.3	TYR-373, GLN-263
IKKβ	4KIK	−6.4	ARG-579, ARG-582

**Table 3:** The SAR of flavonoids (**1–6**), diterpenoids (**7** and **9**), and triterpenoids (**10** and **11**).

**Compound**	**NO Inhibition** **(IC_50_) μM**	**Compound**	**NO Inhibition** **(IC_50_) μM**
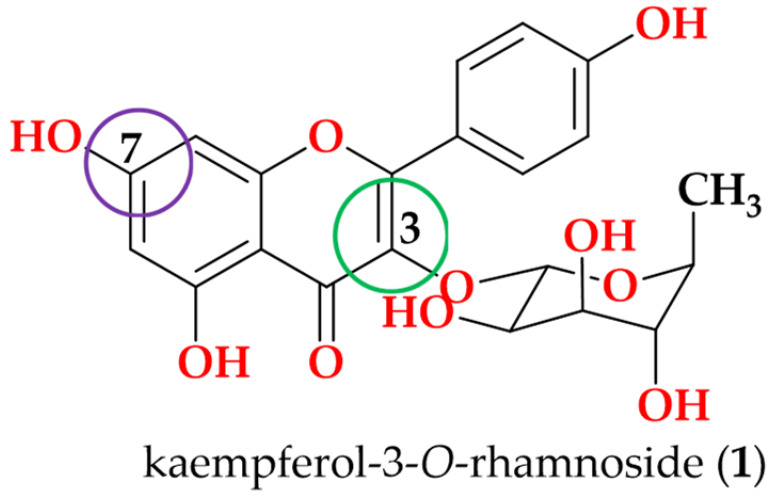	>100	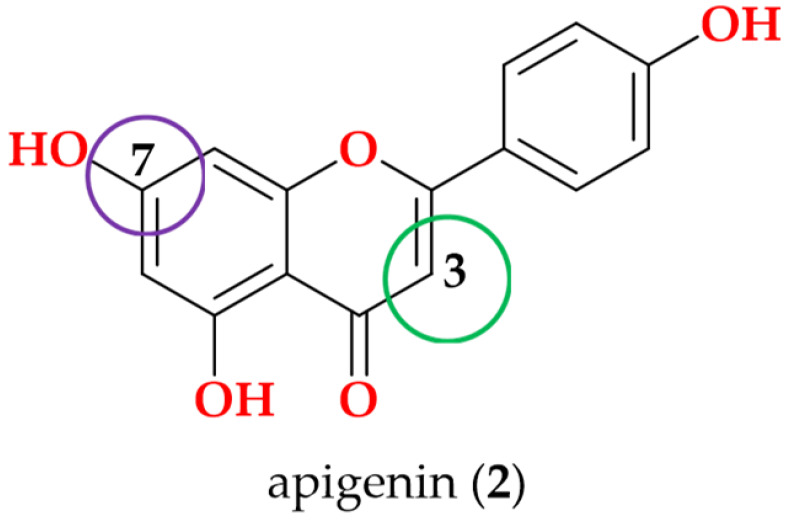	16.4 ± 1.5
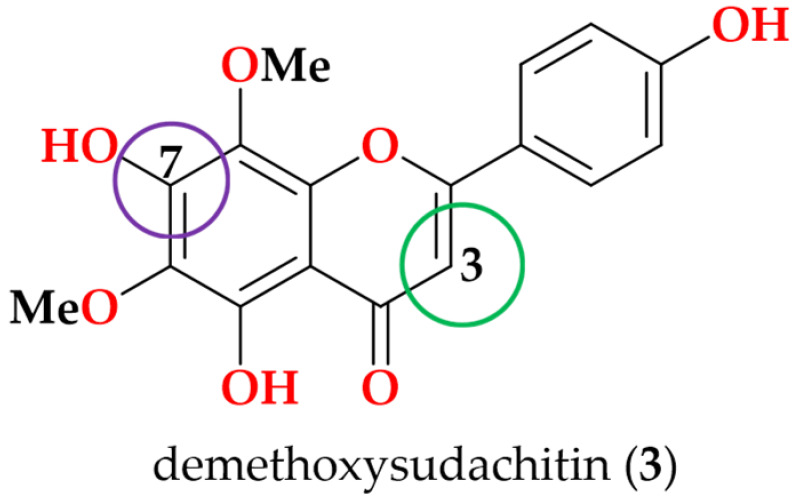	25.6 ± 3.2	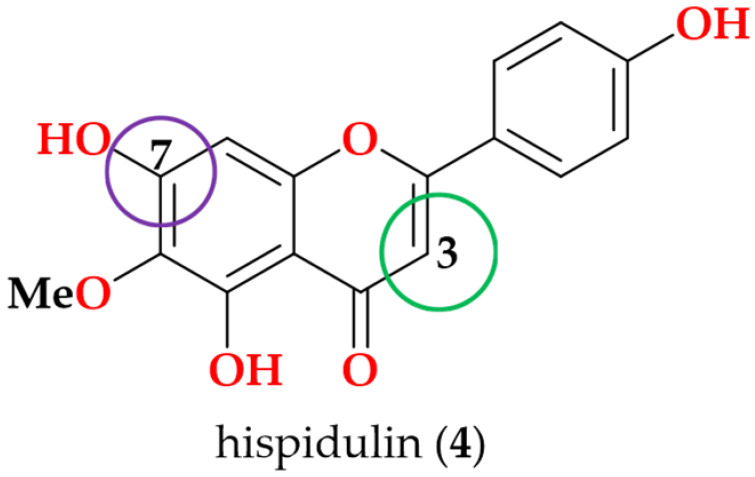	17.6 ± 4.6
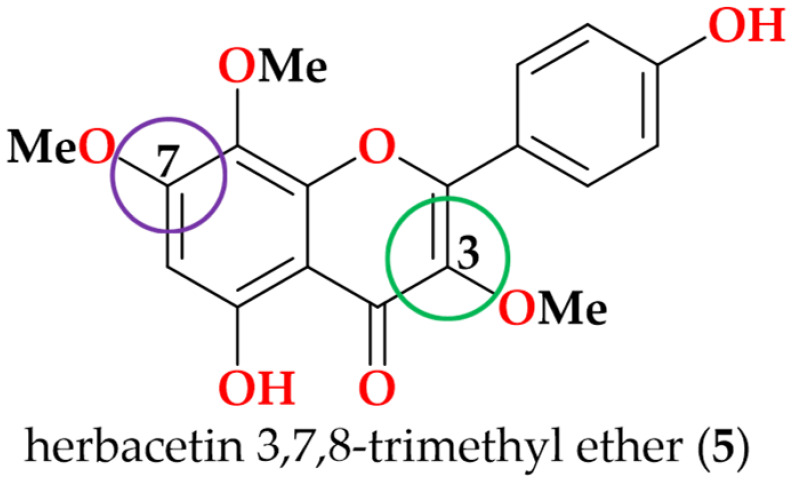	>100	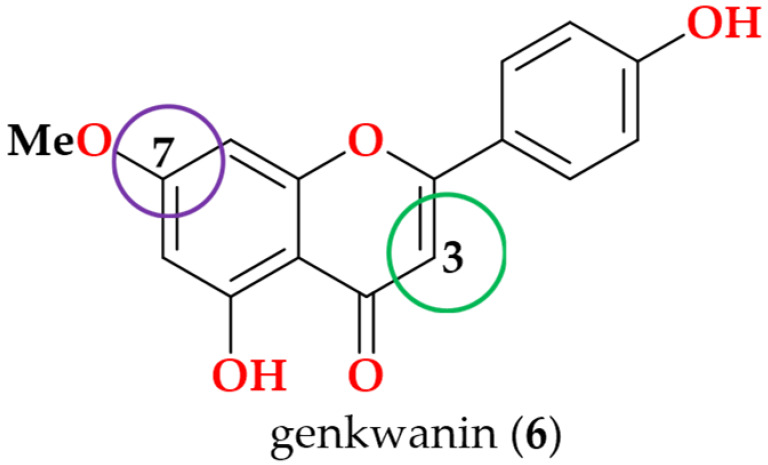	>100
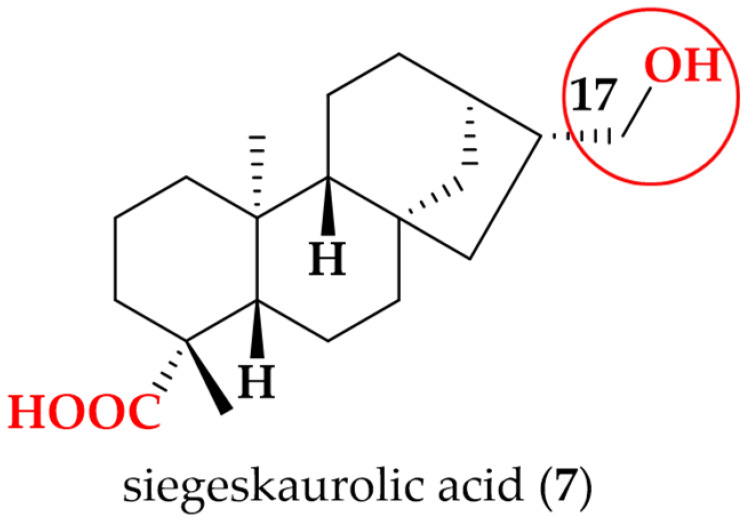	>100	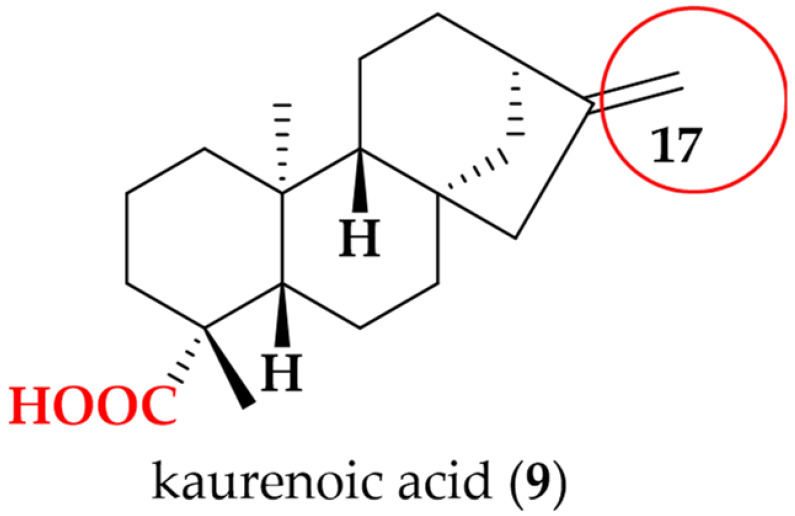	14.1 ± 0.3
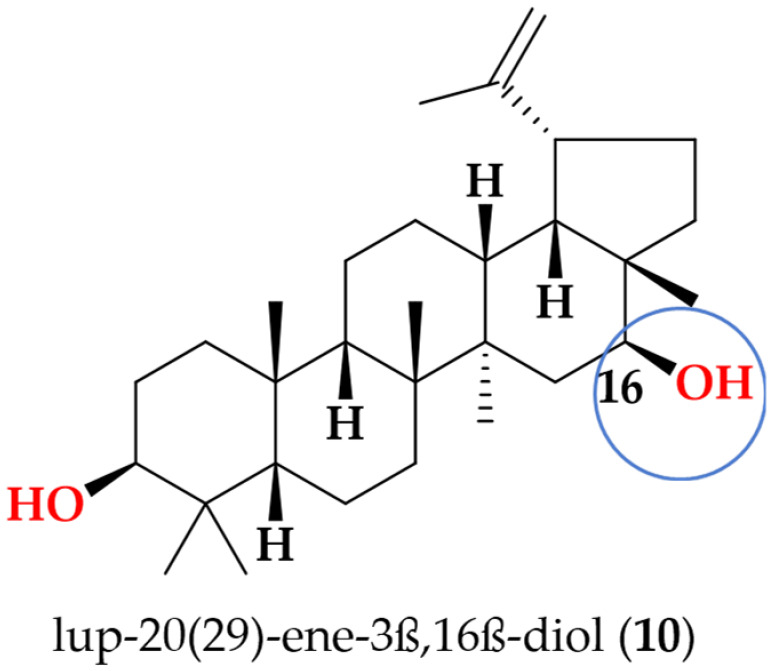	12.2 ± 8.8	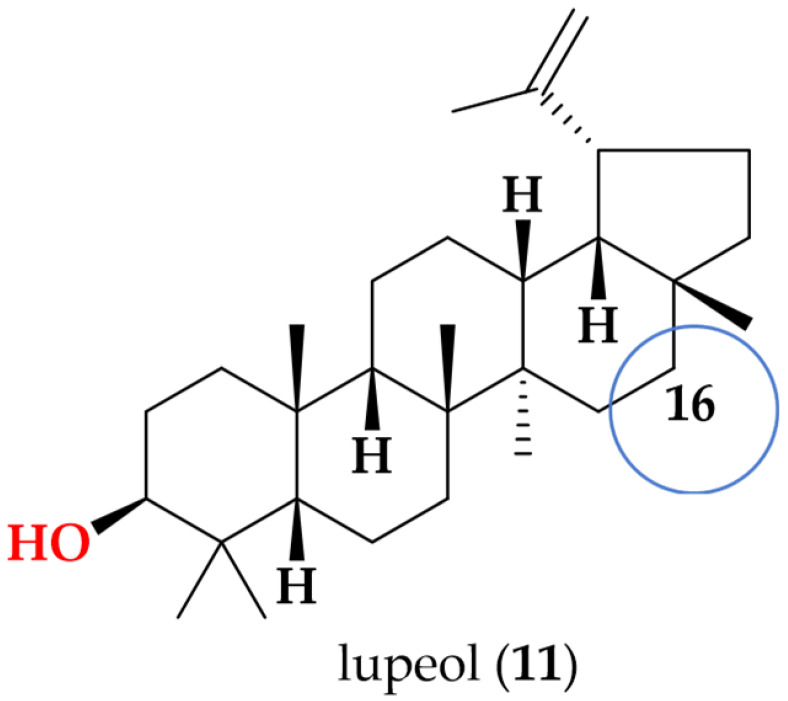	92.8 ± 10.1

Results represent mean ± SD of 3 individual experiments in triplicate.

## Data Availability

The original contributions presented in this study are included in the article and Supplementary Materials. Further inquiries can be directed to the corresponding author.

## Supplementary Materials

The following supporting information can be downloaded at: https://www.mdpi.com/article/10.3390/cimb48050495/s1.
