# Supplementary material for: In Vitro Anti-Inflammatory Activity and Molecular Docking Analysis of Compounds Isolated from Beyeria viscosa
Source: Curr Issues Mol Biol. 2026 May 10;48(5):495. doi: 10.3390/cimb48050495 (PMC13204732; doi:10.3390/cimb48050495)
Supplement: Supplementary file 1 [file cimb-48-00495-s001.zip › cimb-4271372-supplementary.pdf]

Supporting Information

In-vitro anti-inflammatory activity and molecular docking analysis of compounds isolated from *Beyeria viscosa*

Hamza Shahid [1], James P Flood [2], Feng Li [2], Xian Zhou [3], Gerald Münch [1,3] and Ritesh Raju [1,*]

[1] Department of Pharmacology, Western Sydney University, Campbelltown Campus, Sydney, NSW 2751, Australia; 22113146@student.westernsydney.edu.au (H.S)

[2] Western Sydney University, School of Science, Locked Bag 1797, Penrith, NSW 2751, Australia; 19801201@student.westernsydney.edu.au (J.P.F); feng.li@westernsydney.edu.au (F.L)

[3] NICM Health Research Institute, Western Sydney University, Westmead, NSW 2145, Australia; P.Zhou@westernsydney.edu.au (X.Z); G.Muench@westernsydney.edu.au (G.M)

*Correspondence: r.raju@westernsydney.edu.au; Tel.: +61-02-46203799

Table of contents

Figure S1. ¹H NMR spectrum of compound **1** (600 MHz, DMSO-d_6) 4

Figure S2. HRMS of compound **1** 5

Figure S3. ¹H NMR spectrum of compound **2** (600 MHz, DMSO-d_6) 6

Figure S4. HRMS of compound **2** 7

Figure S5. ¹H NMR spectrum of compound 3 (600 MHz, MeOD-d_4).......... 8

Figure S6. HRMS of compound **3** 9

Figure S7. ¹H NMR spectrum of compound **4** (600 MHz, MeOD-d_4).......... 10

Figure S8. HRMS of compound **4** 11

Figure S9. ¹H NMR spectrum of compound **5** (600 MHz, DMSO-d_6) 12

Figure S10. HRMS of compound **5**.......... 13

Table S1. Crystallographic data for compound **5** 14

Table S2. Fractional Atomic Coordinates ($\times10^4$) and Equivalent Isotropic Displacement Parameters ($Å^2\times10^3$) for compound **5**. U_{eq} is defined as 1/3 of the trace of the orthogonalised U_{IJ} tensor.......... 15

Figure S11. 1H NMR spectrum of compound 6 (600 MHz, DMSO-d6).......... 16

Figure S12. HRMS of compound **6** (600 MHz, DMSO-d_6).......... 17

Figure S13. ¹H NMR spectrum of compound **7** (600 MHz, DMSO-d_6) 18

Figure S14. HRMS of compound **7**.......... 19

Figure S15. ¹H NMR spectrum of compound **8** (600 MHz, DMSO-d_6) 20

Figure S16. HRMS of compound **8**.......... 21

Table S3. Crystallographic data for compound **8** 22

Table S4. Fractional Atomic Coordinates (×10^4) and Equivalent Isotropic Displacement Parameters (Å^2×10^3) for compound **8**. U_{eq} is defined as 1/3 of the trace of the orthogonalised U_{IJ} tensor....23

Figure S17. ^{1}H NMR spectrum of compound **9** (600 MHz, MeOD-d_4)....24

Figure S18. HRMS of compound **9**....25

Figure S19. ^{1}H NMR spectrum of compound **10** (600 MHz, MeOD-d_4)....26

Figure S20. HRMS of compound **10**....27

Figure S21. ^{1}H NMR spectrum of compound **11** (600 MHz, $CDCl_3$)....28

Figure S22. HRMS of compound **11**....29

Table S5. *In vitro* anti-inflammatory activity of *B. viscosa* (mature leaves) HPLC fractions in LPS & IFN-γ activated RAW 264.7 macrophages....30

Figure S23. HPLC trace of *Beyeria viscosa* fractions (Fr. 1–19)....31

Figure S24. Concentration response curves of compounds **1–6** in RAW 264.7 macrophages showing inhibition of NO production (IC_{50})**,** cell viability (LC_{50}), and suppression of TNF-α (IC_{50}) under LPS/IFN-γ stimulation. Data are presented as mean ± SD of 3 individual experiments in triplicate. IC_{50} and LC_{50} values were calculated by sigmoidal dose response function and are summarized in in the main text (Table 1)....32

Figure S25. Concentration response curves of compounds **7–11** and curcumin (**+ve control**) in RAW 264.7 macrophages showing inhibition of NO production (IC_{50}), cell viability (LC_{50}), and suppression of TNF-α (IC_{50}) under LPS/IFN-γ stimulation. Data are presented as mean ± SD of 3 individual experiments in triplicate. IC_{50} and LC_{50} values were calculated by sigmoidal dose response function and are summarized in the main text (Table 1). ..33

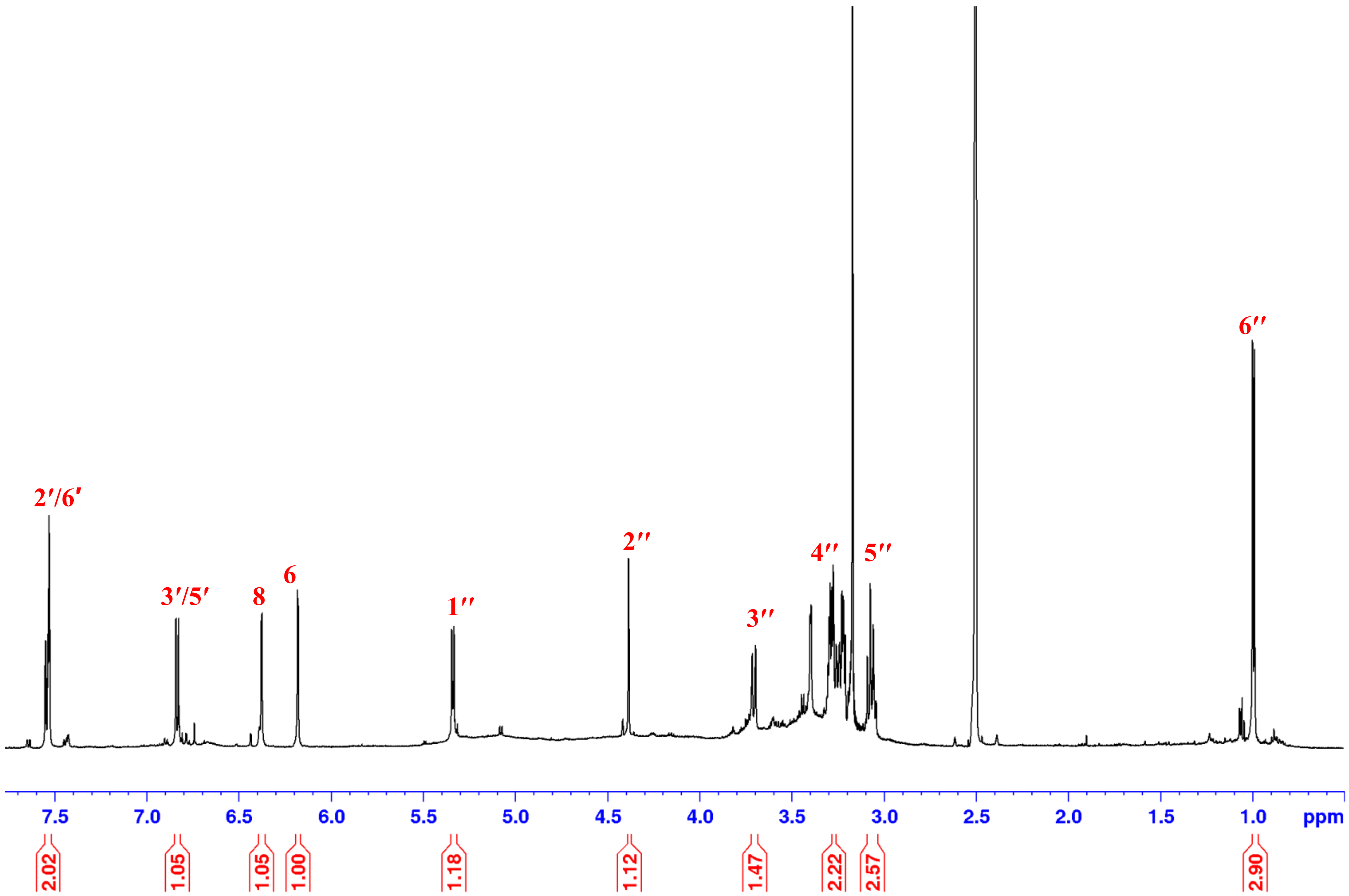

Figure S1. ^{1}H NMR spectrum of compound **1** (600 MHz, DMSO-d_6)

Single Mass Analysis
Tolerance = 250.0 mDa / DBE: min = -1.5, max = 50.0
Element prediction: Off
Number of isotope peaks used for i-FIT = 3
Monoisotopic Mass, Even Electron Ions
1 formula(e) evaluated with 1 results within limits (up to 50 closest results for each mass)
Elements Used:

Mass	Calc. Mass	mDa	PPM	DBE	Formula	i-FIT	i-FIT Norm	Fit Conf %	C	H	O	23Na
455.0964	455.0954	1.0	2.2	11.5	C21 H20 O10 23Na	432.7	n/a	n/a	21	20	10	1

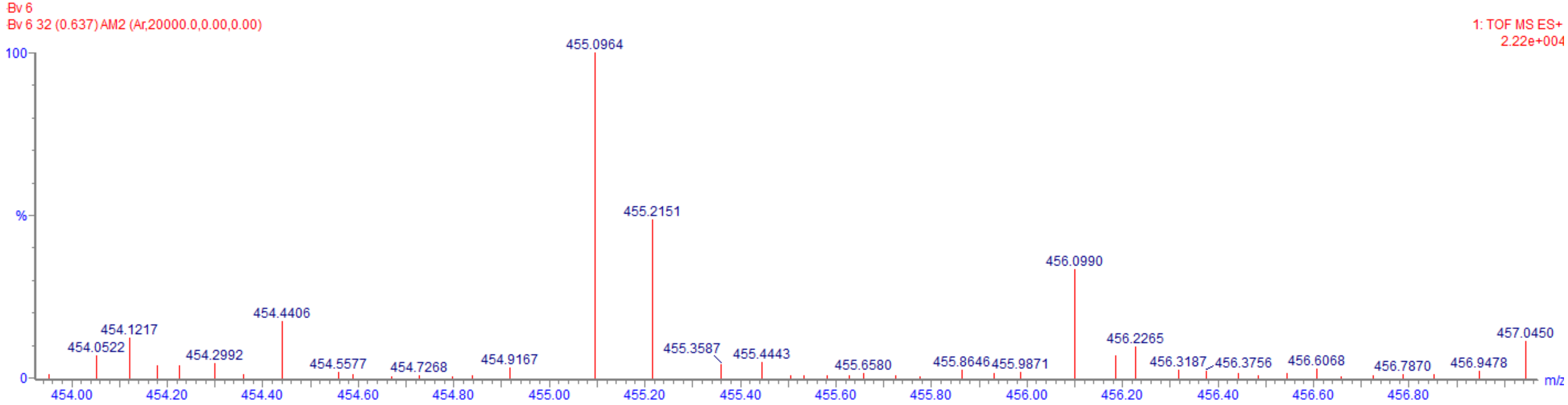

Figure S2. HRMS of compound **1**

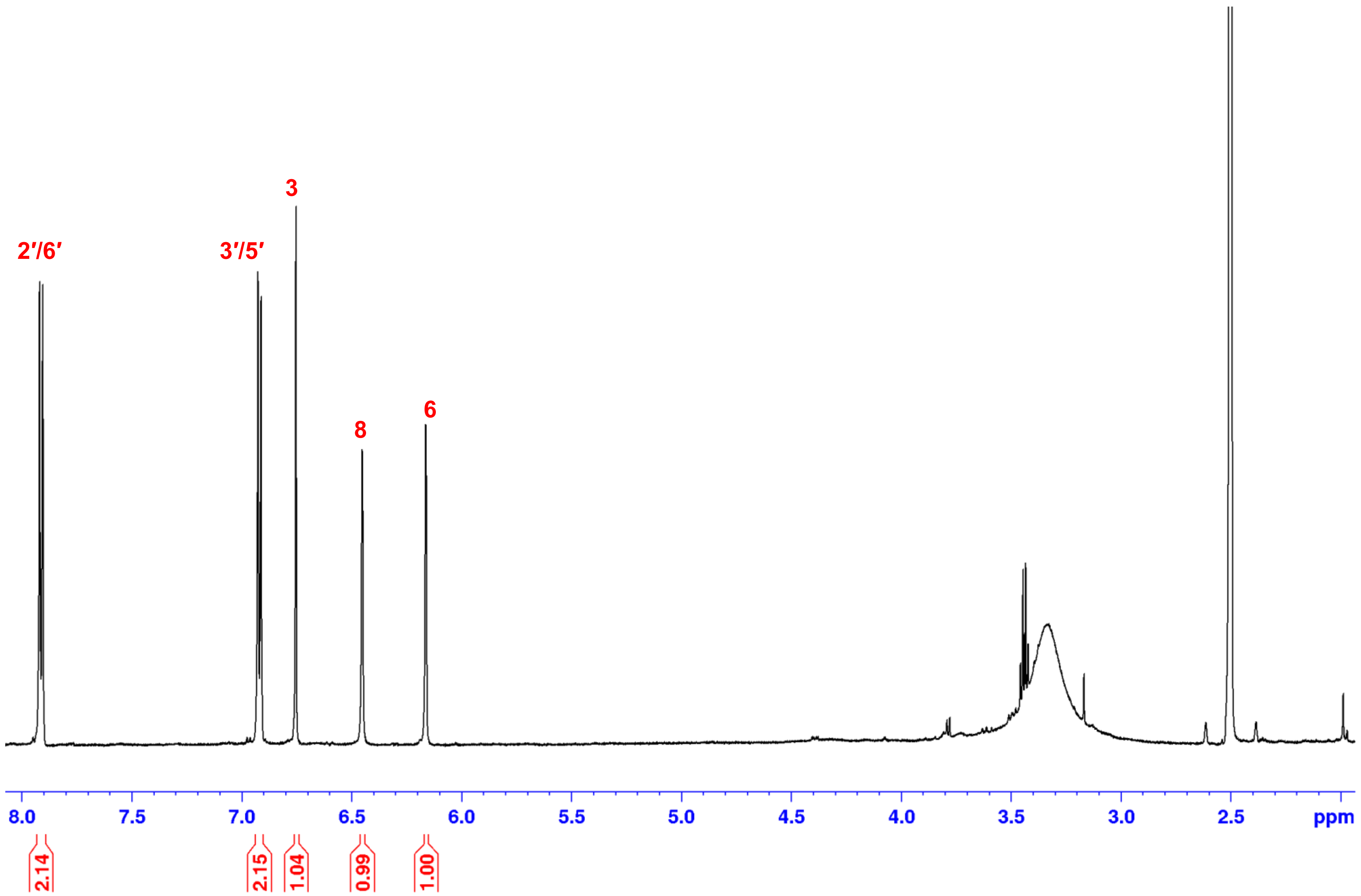

Figure S3. ^{1}H NMR spectrum of compound **2** (600 MHz, DMSO-d_6)

Single Mass Analysis
Tolerance = 250.0 mDa / DBE: min = -1.5, max = 50.0
Element prediction: Off
Number of isotope peaks used for i-FIT = 3
Monoisotopic Mass, Even Electron Ions
10 formula(e) evaluated with 1 results within limits (up to 50 closest results for each mass)
Elements Used:

Mass	Calc. Mass	mDa	PPM	DBE	Formula	i-FIT	i-FIT Norm	Fit Conf %	C	H	O	23Na
293.0426	293.0426	0.0	0.0	10.5	C15 H10 O5 23Na	593.0	n/a	n/a	15	10	5	1

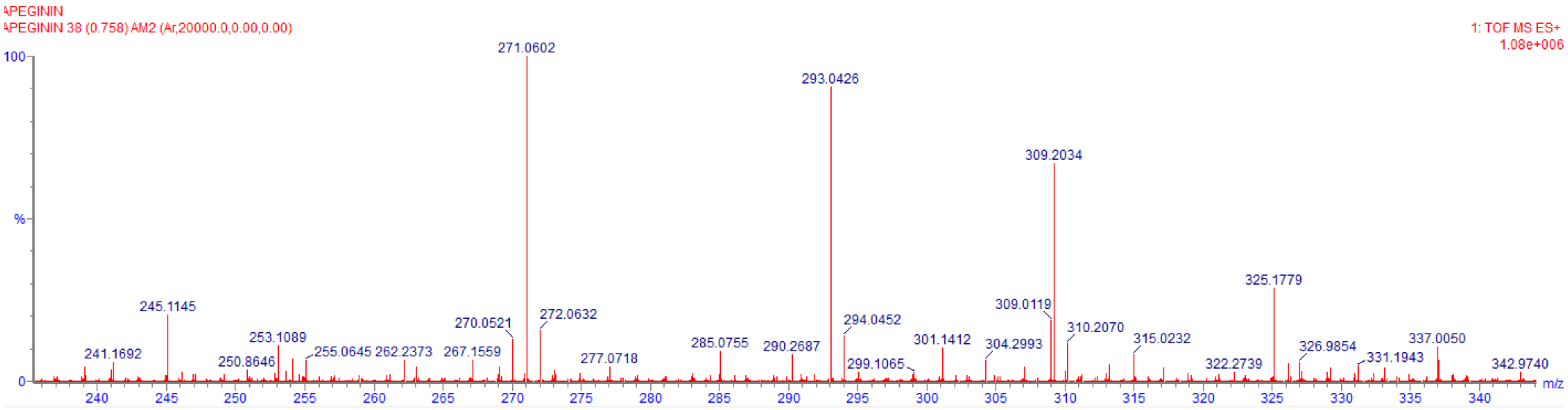

Figure S4. HRMS of compound **2**

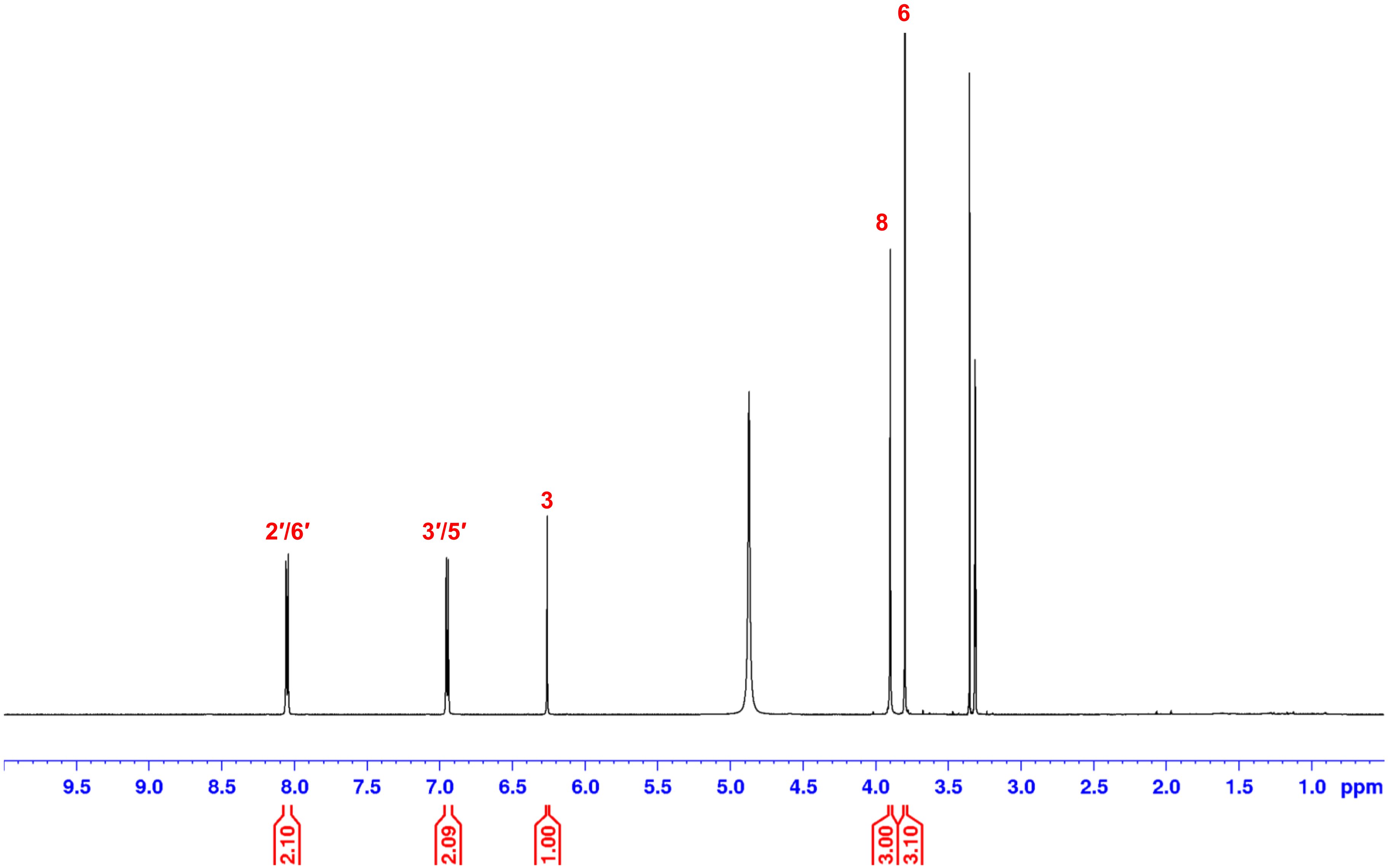

Figure S5. ^{1}H NMR spectrum of compound 3 (600 MHz, MeOD-*d*4)

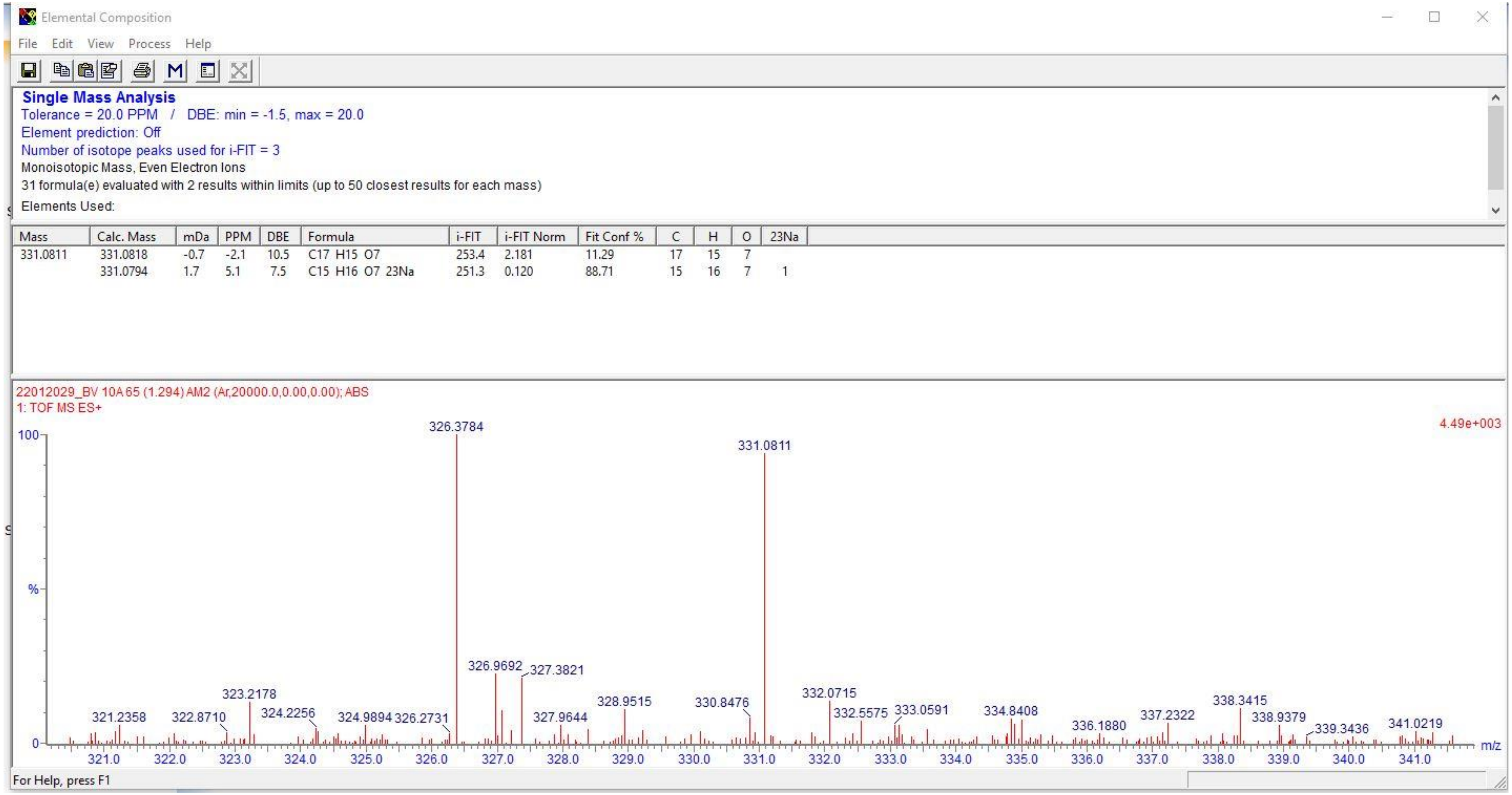

Figure S6. HRMS of compound **3**

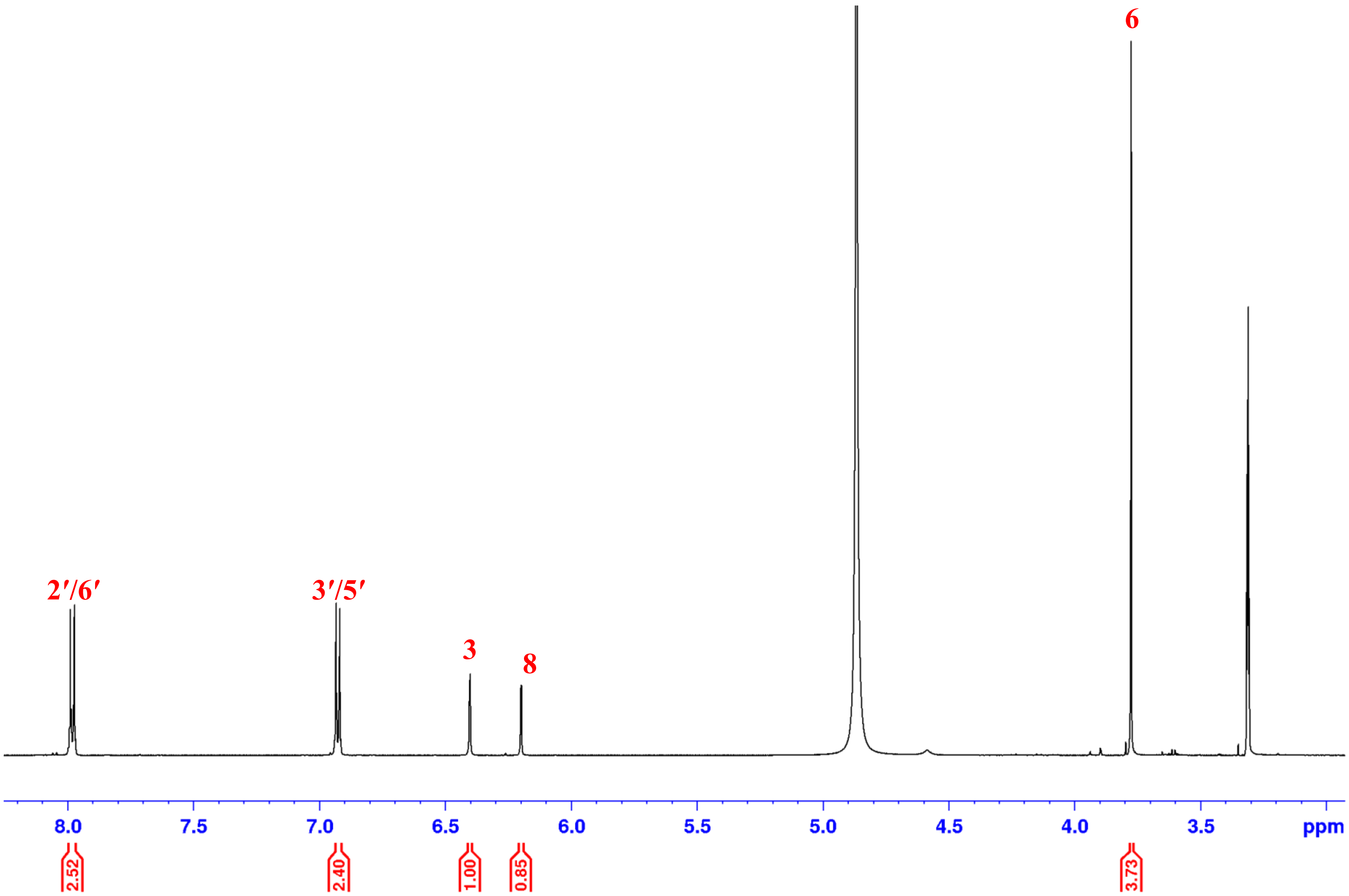

Figure S7. ^{1}H NMR spectrum of compound **4** (600 MHz, MeOD-*d*4)

Single Mass Analysis
Tolerance = 250.0 mDa / DBE: min = -1.5, max = 50.0
Element prediction: Off
Number of isotope peaks used for i-FIT = 3
Monoisotopic Mass, Even Electron Ions
3 formula(e) evaluated with 1 results within limits (up to 50 closest results for each mass)
Elements Used:

Mass	Calc. Mass	mDa	PPM	DBE	Formula	i-FIT	i-FIT Norm	Fit Conf %	C	H	O	23Na
323.0530	323.0532	-0.2	-0.6	10.5	C16 H12 O6 23Na	629.3	n/a	n/a	16	12	6	1

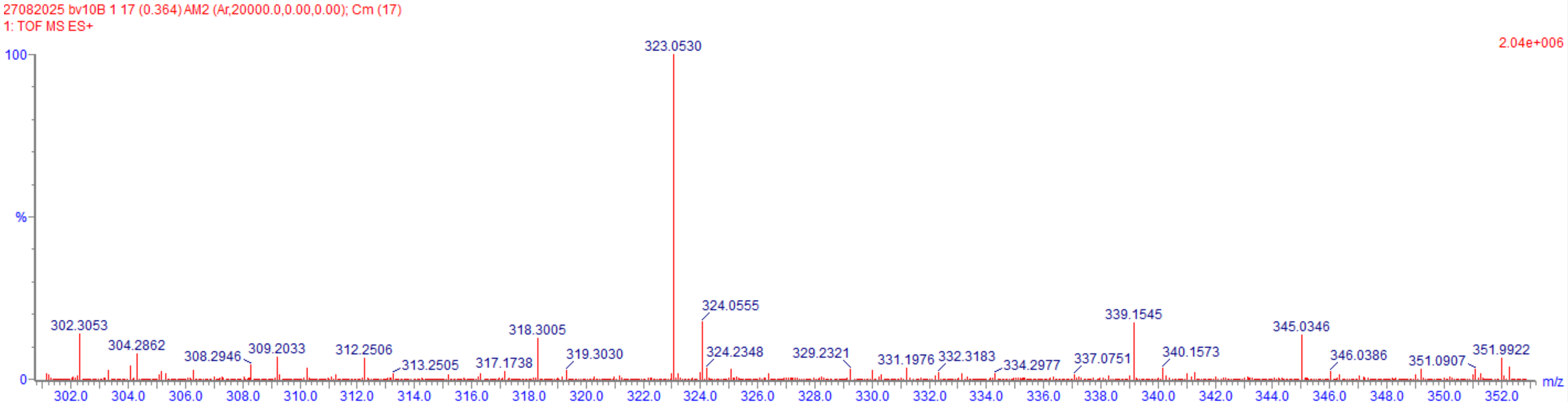

Figure S8. HRMS of compound **4**

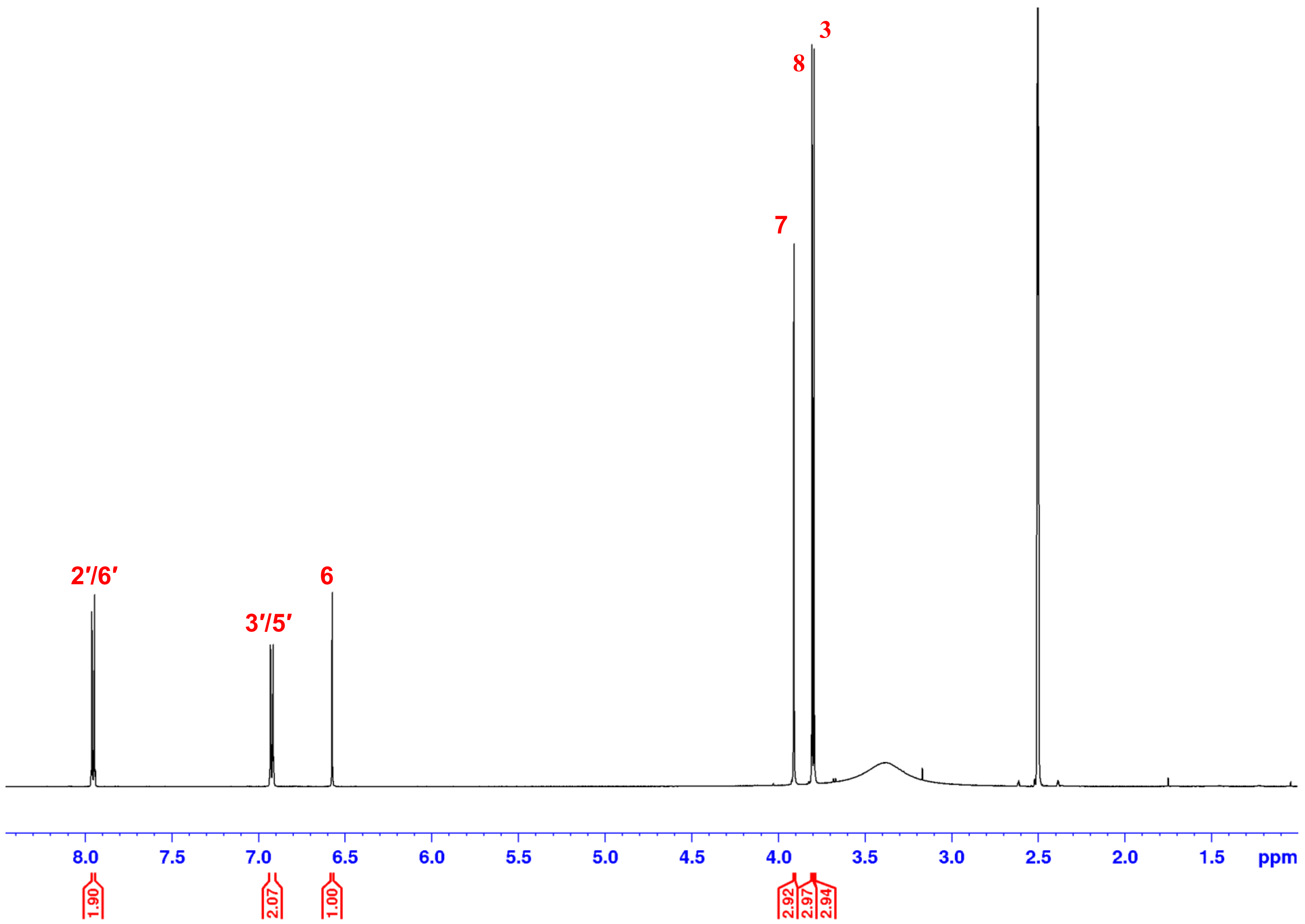

Figure S9. ^{1}H NMR spectrum of compound **5** (600 MHz, DMSO-*d*$_6$)

Single Mass Analysis
Tolerance = 100.0 PPM / DBE: min = -1.5, max = 50.0
Element prediction: Off
Number of isotope peaks used for i-FIT = 3
Monoisotopic Mass, Even Electron Ions
7 formula(e) evaluated with 1 results within limits (all results (up to 1000) for each mass)
Elements Used:

Mass	Calc. Mass	mDa	PPM	DBE	Formula	i-FIT	i-FIT Norm	Fit Conf %	C	H	O
345.0979	345.0974	0.5	1.4	10.5	C18 H17 O7	580.3	n/a	n/a	18	17	7

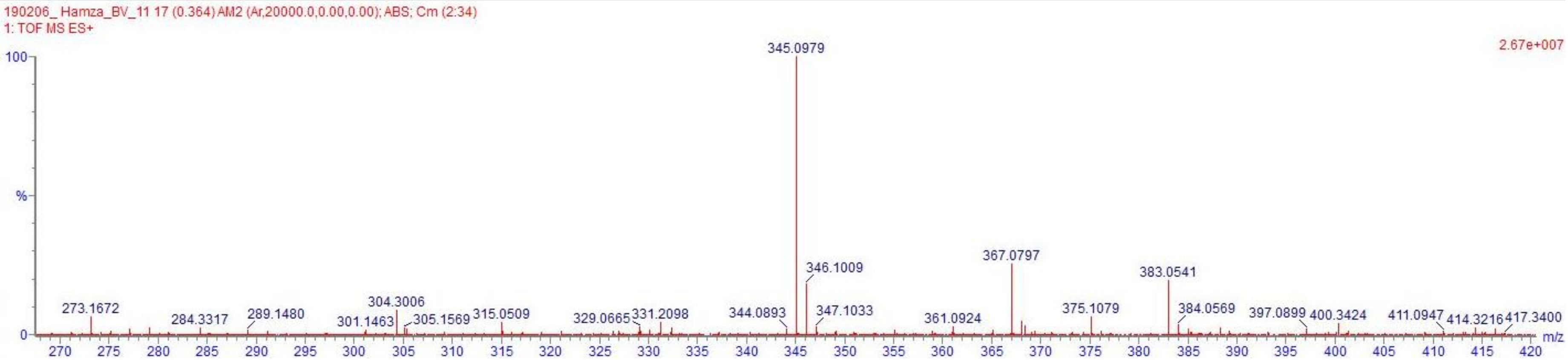

Figure S10. HRMS of compound **5**

Table S1. Crystallographic data for compound **5**

**Empirical formula**	$C_{18}H_{16}O_7$
**Formula weight**	344.31
**Temperature/K**	293(2)
**Crystal system**	monoclinic
**Space group**	*P2₁/n*
**a/Å**	3.9900(8)
**b/Å**	18.250(4)
**c/Å**	20.820(4)
**α/°**	90
**β/°**	94.16(3)
**γ/°**	90
**Volume/Å^3**	1512.1(5)
**Z**	4
**ρ_{calc}g/cm^3**	1.512
**μ/mm^{-1}**	0.118
**F(000)**	720.0
**Crystal size/mm^3**	0.02 × 0.01 × 0.01
**Radiation**	MoKα (λ = 0.71073)
**2Θ range for data collection/°**	2.97 to 57.298
**Index ranges**	$-5 \le h \le 5$, $-24 \le k \le 24$, $-27 \le l \le 27$
**Reflections collected**	27505
**Independent reflections**	3373 [R_{int} = 0.0455, R_{sigma} = 0.0249]
**Data/restraints/parameters**	3373/0/247
**Goodness-of-fit on F^2**	1.029
**Final R indexes [I>=2σ (I)]**	R_1 = 0.0539, wR_2 = 0.1568
**Final R indexes [all data]**	R_1 = 0.0654, wR_2 = 0.1825
**Largest diff. peak/hole / e Å^{-3}**	0.44/-0.33
**Flack parameter**	-

Table S2. Fractional Atomic Coordinates (×10[4]) and Equivalent Isotropic Displacement Parameters (Å^2×10^3) for compound **5**. U_{eq} is defined as 1/3 of the trace of the orthogonalised U_{IJ} tensor

Atom	*x*	*y*	*z*	U(eq)
O001	7749(3)	2881.9(6)	5891.1(5)	31.5(3)
O002	11153(3)	2503.6(6)	7519.3(5)	33.5(3)
O003	1767(3)	1537.7(6)	4288.4(5)	34.5(3)
O004	5419(3)	2719.9(6)	4648.1(5)	32.7(3)
O005	7786(3)	1223.0(6)	7197.3(5)	35.2(3)
O006	4056(3)	448.9(6)	6369.5(6)	36.5(3)
O007	13236(3)	5948.1(6)	6708.3(6)	39.2(3)
C008	6168(4)	2239.9(8)	5720.4(7)	30.5(4)
C009	10468(4)	3750.0(8)	6565.3(7)	31.0(4)
C00A	2834(4)	964.7(8)	5339.3(7)	32.3(4)
C00B	4818(4)	2189.1(8)	5093.3(7)	30.8(4)
C00C	5998(4)	1667.8(8)	6165.7(7)	30.5(4)
C00D	7707(4)	1741.0(8)	6795.8(7)	31.5(4)
C00E	3074(4)	1546.9(8)	4903.9(7)	31.0(4)
C00F	4289(4)	1021.9(8)	5959.3(7)	31.6(4)
C00G	9338(4)	2435.8(8)	6938.9(7)	30.7(4)
C00H	9224(4)	2993.1(8)	6494.0(7)	30.5(4)
C00I	12414(4)	5229.7(8)	6681.9(7)	32.8(4)
C00J	9652(4)	4255.5(8)	6068.7(7)	33.3(4)
C00K	12322(4)	4006.6(8)	7115.0(7)	34.1(4)
C00L	13266(4)	4735.1(8)	7177.2(7)	34.1(4)
C00M	10618(4)	4980.3(9)	6124.7(8)	35.2(4)
C00N	-33(4)	891.6(9)	4065.6(8)	35.2(4)
C00O	9167(4)	2622.0(9)	8063.5(8)	37.8(4)
C00P	3073(5)	3315.7(10)	4598.5(9)	42.6(4)

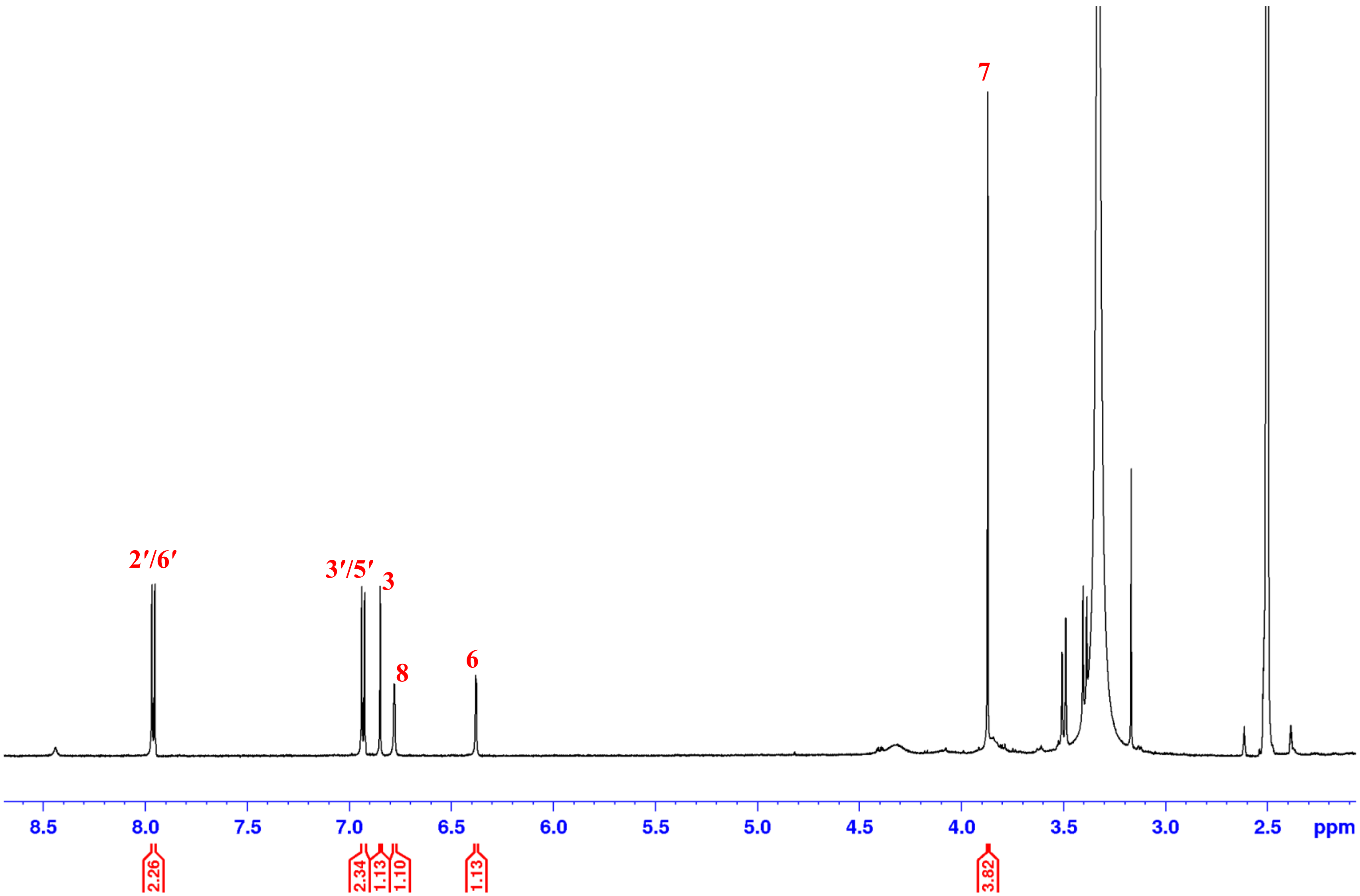

Figure S11. 1H NMR spectrum of compound 6 (600 MHz, DMSO-d6)

Single Mass Analysis
Tolerance = 100.0 PPM / DBE: min = -1.5, max = 50.0
Element prediction: Off
Number of isotope peaks used for i-FIT = 3
Monoisotopic Mass, Even Electron Ions
5 formula(e) evaluated with 1 results within limits (all results (up to 1000) for each mass)
Elements Used:

Mass	Calc. Mass	mDa	PPM	DBE	Formula	i-FIT	i-FIT Norm	Fit Conf %	C	H	O
285.0767	285.0763	0.4	1.4	10.5	C16 H13 O5	758.1	n/a	n/a	16	13	5

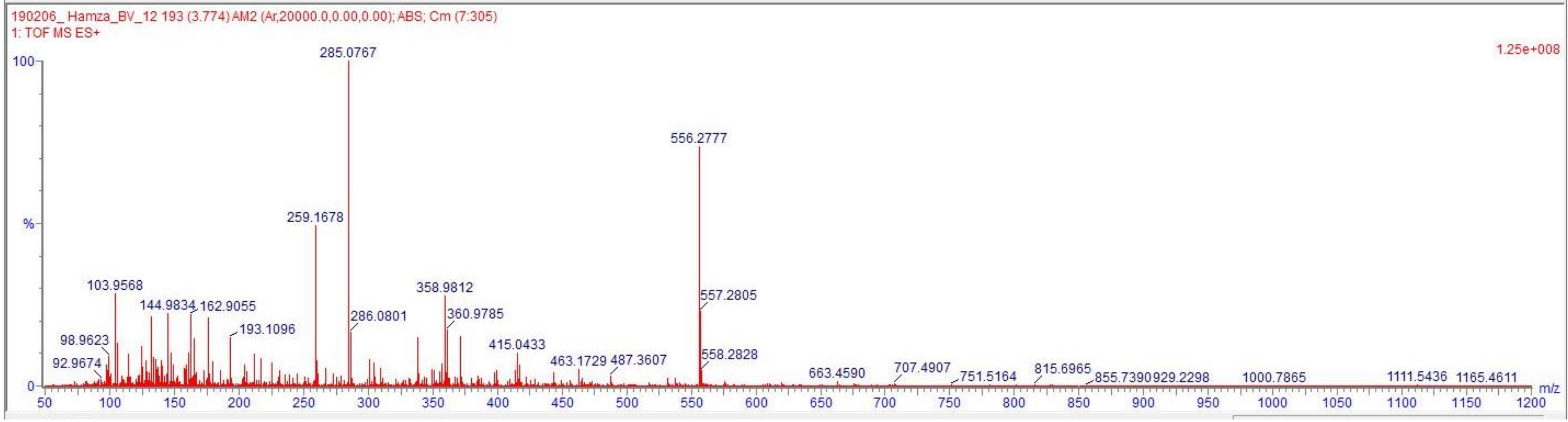

Figure S12. HRMS of compound **6** (600 MHz, DMSO-d_6)

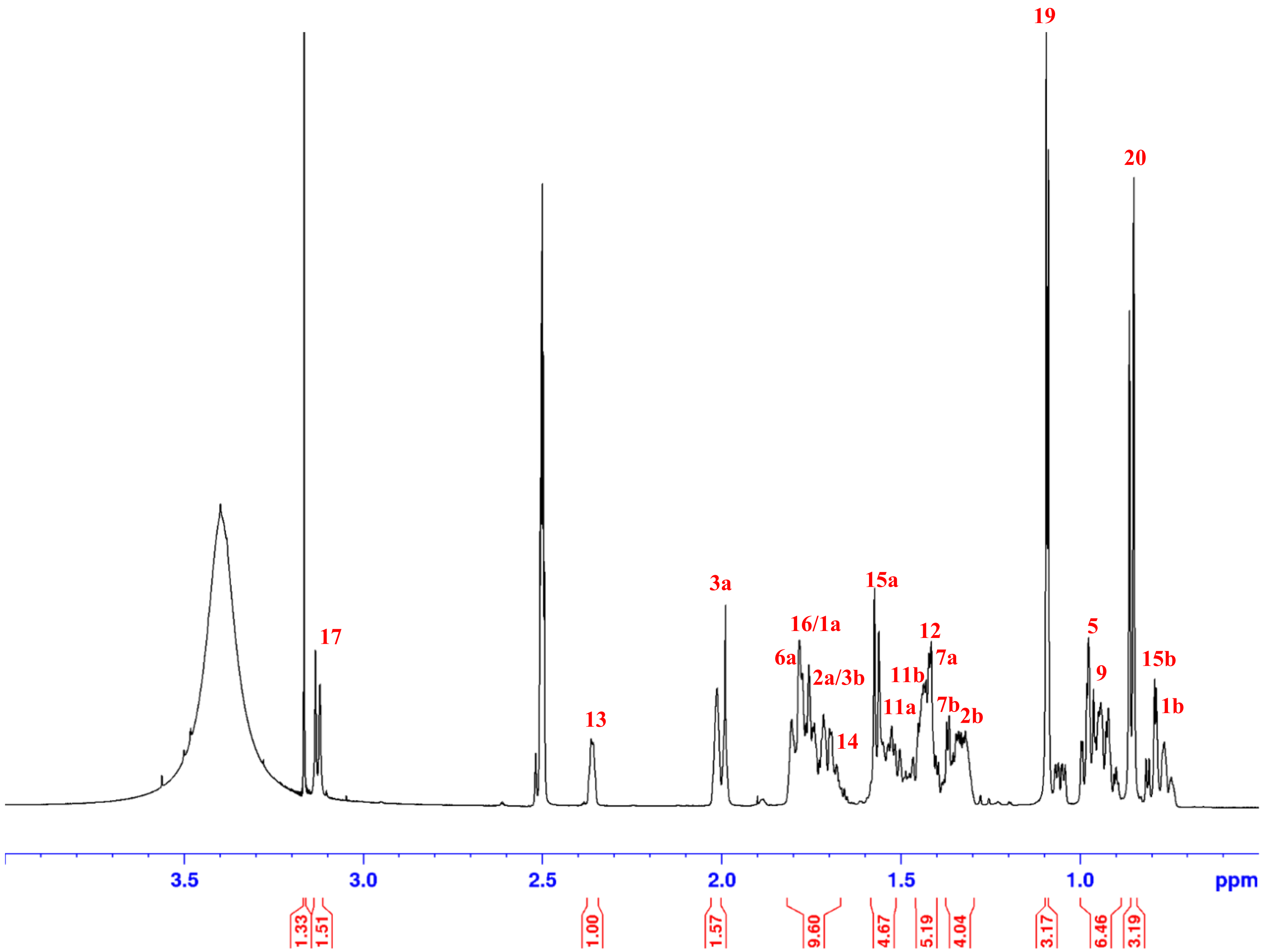

Figure S13. ^{1}H NMR spectrum of compound **7** (600 MHz, DMSO-d_6)

Single Mass Analysis
Tolerance = 20.0 PPM / DBE: min = -1.5, max = 50.0
Element prediction: Off
Number of isotope peaks used for i-FIT = 3
Monoisotopic Mass, Even Electron Ions
2 formula(e) evaluated with 1 results within limits (up to 50 closest results for each mass)
Elements Used:

Mass	Calc. Mass	mDa	PPM	DBE	Formula	i-FIT	i-FIT Norm	Fit Conf %	C	H	O	23Na
343.2244	343.2249	-0.5	-1.5	4.5	C20 H32 O3 23Na	19.3	n/a	n/a	20	32	3	1

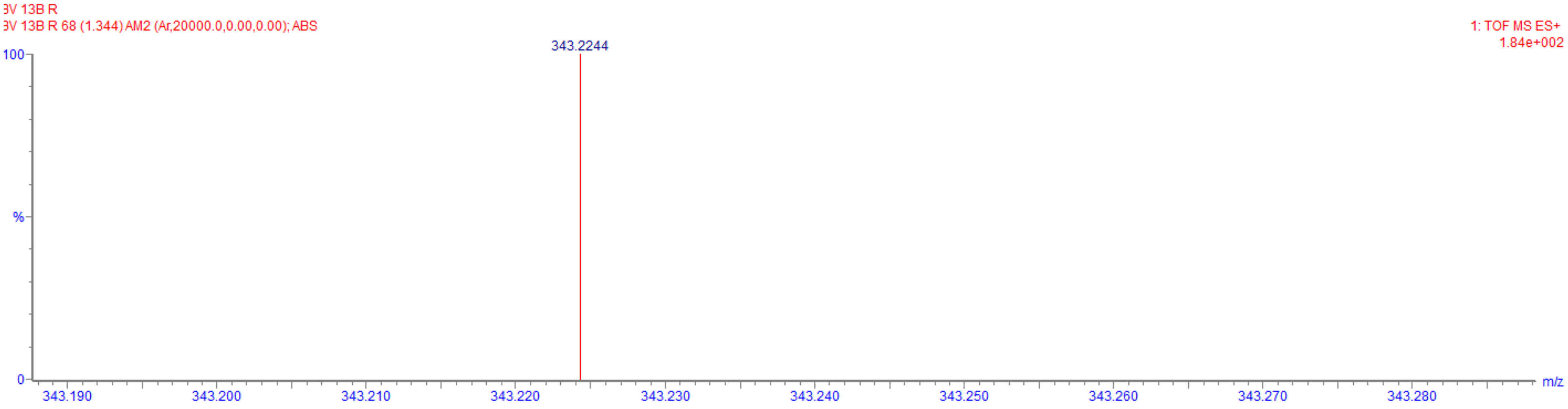

Figure S14. HRMS of compound **7**

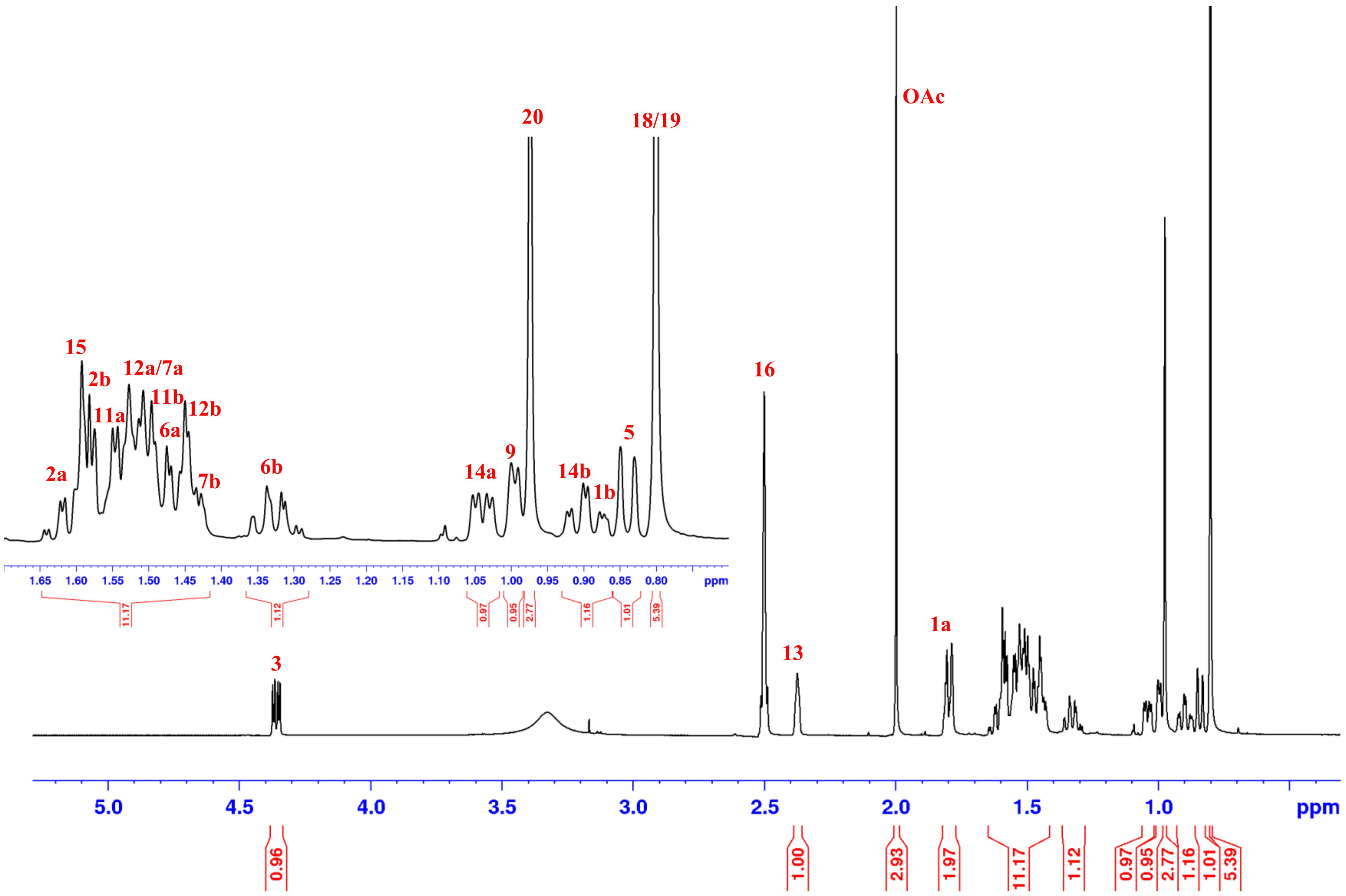

Figure S15. ^{1}H NMR spectrum of compound **8** (600 MHz, DMSO-*d*6)

Single Mass Analysis
Tolerance = 20.0 PPM / DBE: min = -1.5, max = 50.0
Element prediction: Off
Number of isotope peaks used for i-FIT = 3
Monoisotopic Mass, Even Electron Ions
1 formula(e) evaluated with 1 results within limits (up to 50 closest results for each mass)
Elements Used:

Mass	Calc. Mass	mDa	PPM	DBE	Formula	i-FIT	i-FIT Norm	Fit Conf %	C	H	O	23Na
363.2548	363.2535	1.3	3.6	5.5	C22 H35 O4	40.4	n/a	n/a	22	35	4	

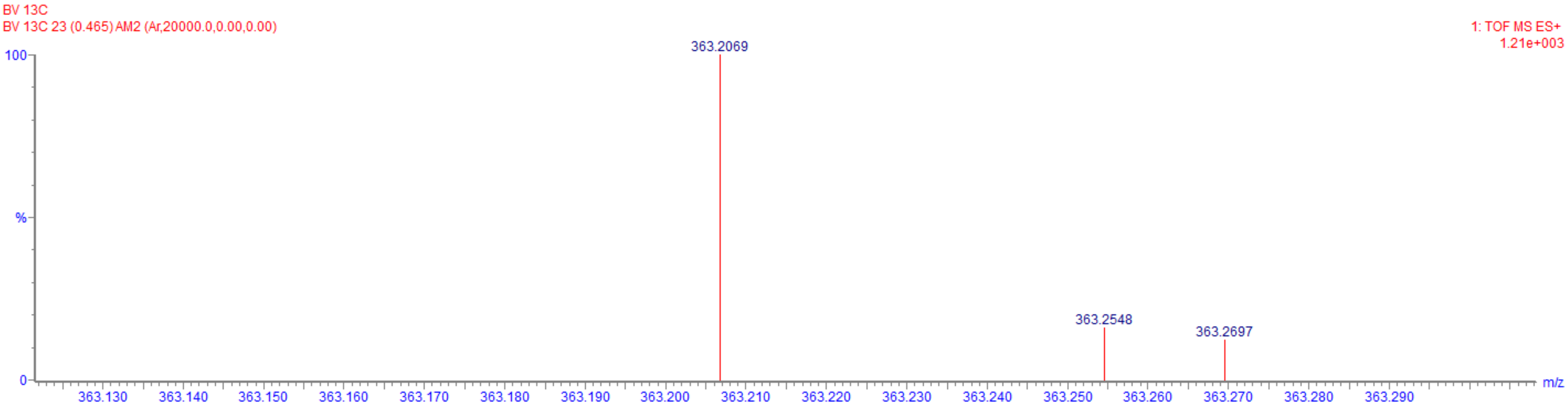

Figure S16. HRMS of compound **8**

Table S3. Crystallographic data for compound **8**

**Empirical formula**	$C_{22}H_{33}O_4$
**Formula weight**	361.48
**Temperature/K**	293(2)
**Crystal system**	monoclinic
**Space group**	*C2*
**a/Å**	12.940(3)
**b/Å**	6.3400(13)
**c/Å**	25.060(5)
**α/°**	90
**β/°**	104.57(3)
**γ/°**	90
**Volume/Å^3**	1989.8(7)
**Z**	4
**ρ_{calc}g/cm^3**	1.207
**μ/mm^{-1}**	0.081
**F(000)**	788.0
**Crystal size/mm^3**	0.02 × 0.02 × 0.01
**Radiation**	MoKα (λ = 0.71073)
**2Θ range for data collection/°**	1.68 to 57.39
**Index ranges**	$-16 \le h \le 14$, $-8 \le k \le 8$, $-33 \le l \le 32$
**Reflections collected**	17732
**Independent reflections**	4234 [R_{int} = 0.0819, R_{sigma} = 0.0726]
**Data/restraints/parameters**	4234/10/251
**Goodness-of-fit on F^2**	1.037
**Final R indexes [I>=2σ (I)]**	R_1 = 0.1053, wR_2 = 0.2264
**Final R indexes [all data]**	R_1 = 0.1206, wR_2 = 0.2333
**Largest diff. peak/hole / e Å^{-3}**	0.36/-0.41
**Flack parameter**	0(5)

Table S4. Fractional Atomic Coordinates (×10[4]) and Equivalent Isotropic Displacement Parameters (Å[2]×10[3]) for compound **8**. U_{eq} is defined as 1/3 of the trace of the orthogonalised U_{IJ} tensor

Atom	x	y	z	U(eq)
O001	-2330(4)	-5912(10)	-9089(2)	67.1(15)
O002	-1815(4)	-9309(9)	-9004(3)	73.3(16)
O003	-3548(5)	-2047(13)	-4878(3)	73(2)
O004	-4897(5)	-2030(16)	-5630(2)	92(3)
C005	-2540(5)	-3512(13)	-7497(3)	58.5(19)
C006	-3604(5)	-3652(14)	-6728(3)	61(2)
C007	-3513(6)	-5092(11)	-8489(4)	58.1(19)
C008	-3462(5)	-4897(12)	-7862(3)	57.1(18)
C009	-1954(6)	-7669(13)	-9236(4)	67(2)
C00A	-3809(19)	-1650(90)	-5401(16)	55(4)
C00B	-1483(5)	-4378(13)	-7595(3)	61.1(19)
C00C	-2397(5)	-5830(14)	-8514(3)	59.2(18)
C00D	-4483(5)	-4776(14)	-7140(3)	60.0(19)
C00E	-1495(6)	-4446(15)	-8205(3)	64(2)
C00F	-2526(5)	-3912(12)	-6889(3)	58.7(19)
C00G	-4536(5)	-4322(15)	-7743(3)	61.8(19)
C00H	-3039(6)	-774(16)	-6107(3)	67(2)
C00I	-4296(6)	-6836(15)	-8737(3)	69(2)
C00J	-1934(6)	-724(16)	-6220(4)	72(2)
C00K	-3492(6)	-4521(18)	-6139(3)	74(2)
C00L	-3855(6)	-3051(14)	-8825(3)	65(2)
C00M	-2616(6)	-1161(14)	-7643(3)	66(2)
C00N	-1640(6)	-2829(15)	-6446(3)	67(2)
C00O	-3124(6)	-2686(16)	-5734(3)	69(2)
C00P	-1788(7)	-7442(18)	-9824(3)	78(2)
C00Q	-3864(6)	-1321(16)	-6652(3)	70(2)
O0	-4470(50)	-540(100)	-5430(20)	92(3)
C1	-3937(11)	-2260(40)	-5408(4)	55(4)
O1	-3590(50)	-3040(110)	-4871(15)	73(2)

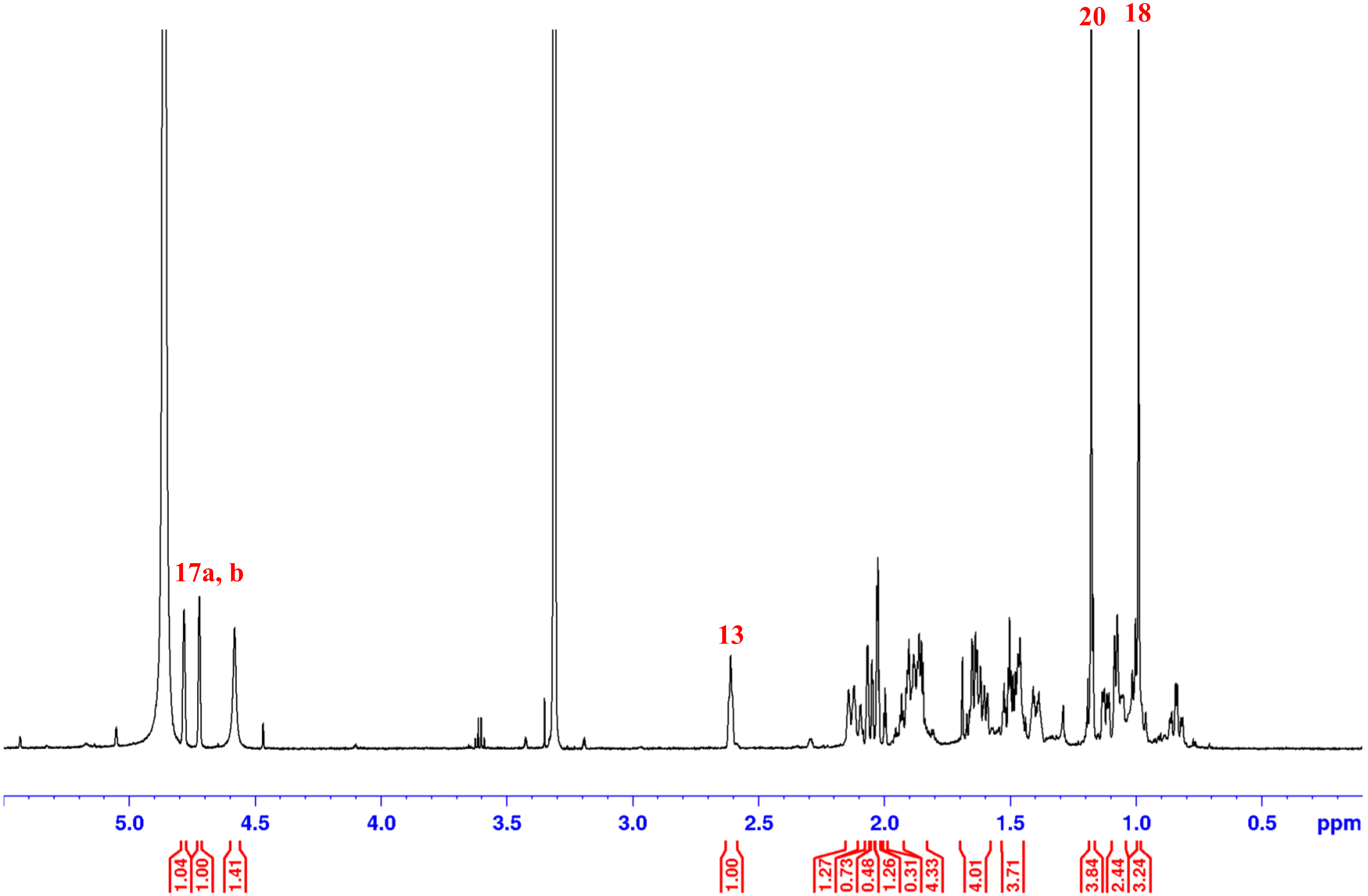

Figure S17. ^{1}H NMR spectrum of compound **9** (600 MHz, MeOD-*d*4)

Single Mass Analysis
Tolerance = 250.0 mDa / DBE: min = -1.5, max = 50.0
Element prediction: Off
Number of isotope peaks used for i-FIT = 3
Monoisotopic Mass, Even Electron Ions
2 formula(e) evaluated with 1 results within limits (up to 50 closest results for each mass)
Elements Used:

Mass	Calc. Mass	mDa	PPM	DBE	Formula	i-FIT	i-FIT Norm	Fit Conf %	C	H	O	23Na
325.2148	325.2143	0.5	1.5	5.5	C20 H30 O2 23Na	486.9	n/a	n/a	20	30	2	1

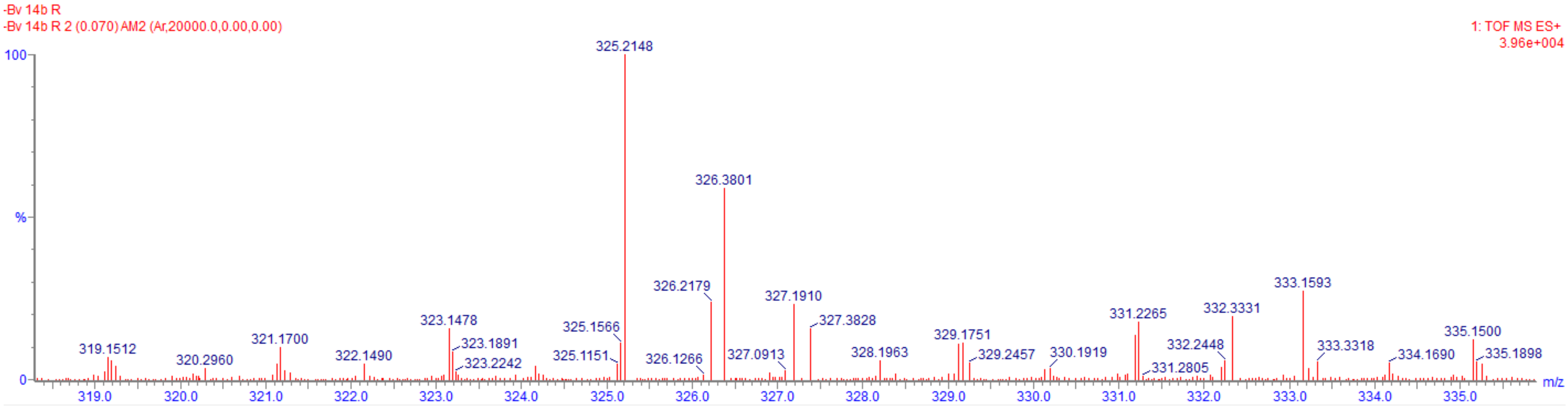

Figure S18. HRMS of compound **9**

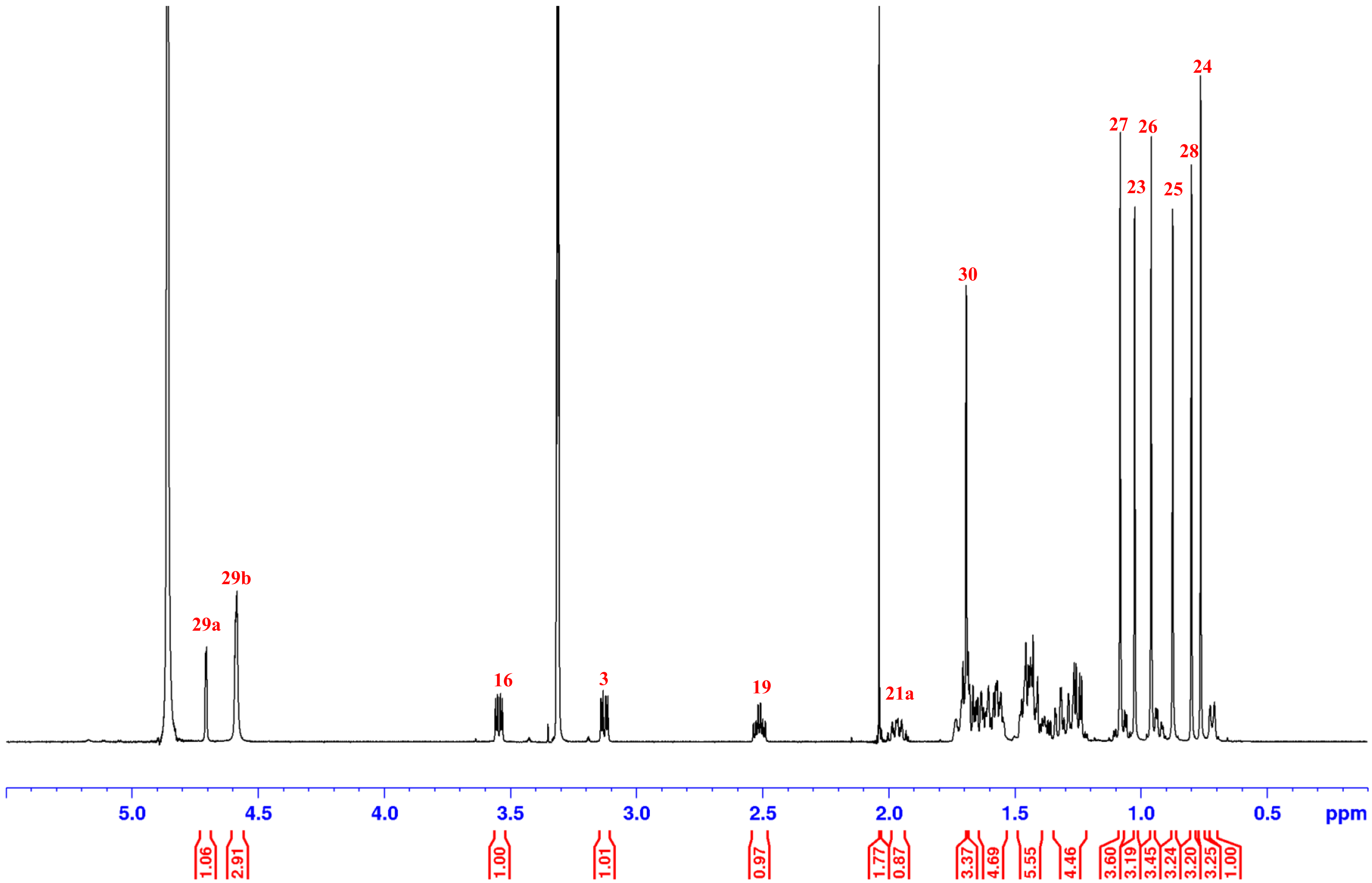

Figure S19. ^{1}H NMR spectrum of compound **10** (600 MHz, MeOD-d_4)

Single Mass Analysis
Tolerance = 20.0 PPM / DBE: min = -1.5, max = 50.0
Element prediction: Off
Number of isotope peaks used for i-FIT = 3
Monoisotopic Mass, Even Electron Ions
10 formula(e) evaluated with 1 results within limits (up to 50 closest results for each mass)
Elements Used:

Mass	Calc. Mass	mDa	PPM	DBE	Formula	i-FIT	i-FIT Norm	Fit Conf %	C	H	O	23Na
443.3889	443.3889	0.0	0.0	5.5	C30 H51 O2	40.6	n/a	n/a	30	51	2	

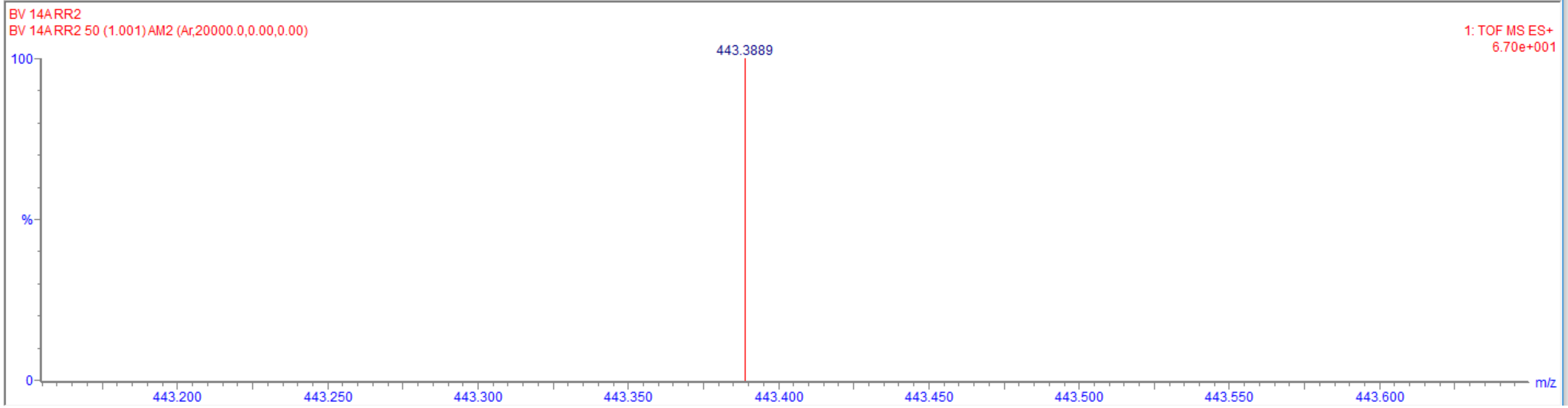

Figure S20. HRMS of compound **10**

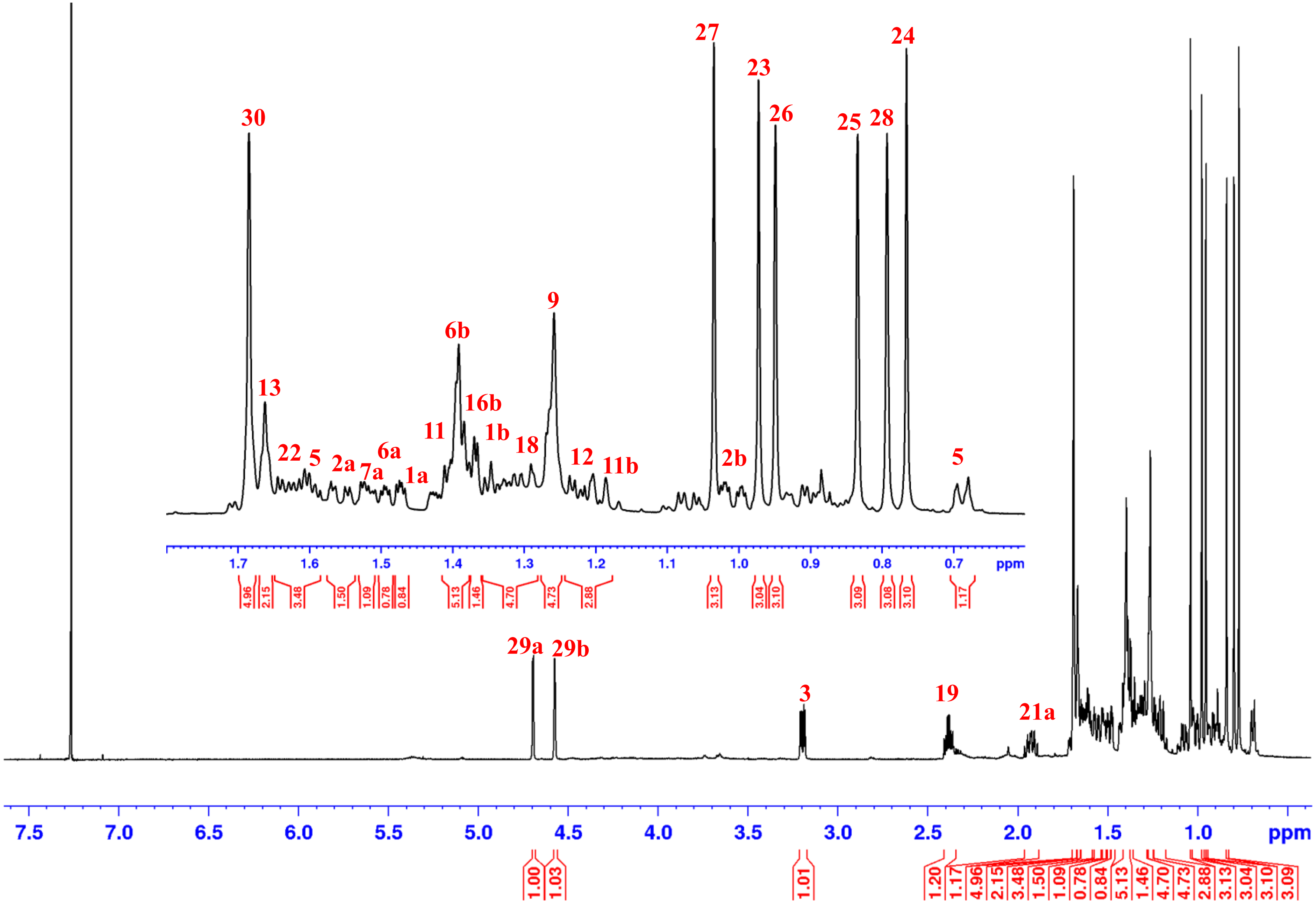

Figure S21. ^{1}H NMR spectrum of compound **11** (600 MHz, $CDCl_3$)

Single Mass Analysis
Tolerance = 20.0 PPM / DBE: min = -1.5, max = 50.0
Element prediction: Off
Number of isotope peaks used for i-FIT = 3
Monoisotopic Mass, Even Electron Ions
9 formula(e) evaluated with 1 results within limits (up to 50 closest results for each mass)
Elements Used:

Mass	Calc. Mass	mDa	PPM	DBE	Formula	i-FIT	i-FIT Norm	Fit Conf %	C	H	O	23Na
427.3941	427.3940	0.1	0.2	5.5	C30 H51 O	15.6	n/a	n/a	30	51	1	

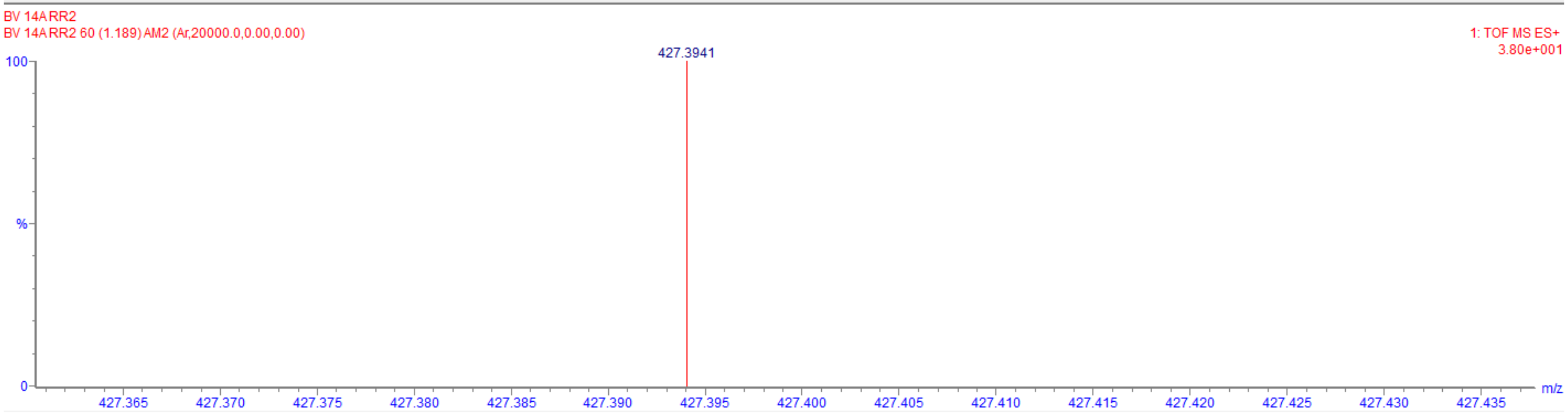

Figure S22. HRMS of compound **11**

Table S5. *In vitro* anti-inflammatory activity of *B. viscosa* (mature leaves) HPLC fractions in LPS & IFN-γ activated RAW 264.7 macrophages

**Fractions**	**Inhibition of NO Production (IC_{50}) µg/mL**	**Cell viability (LC_{50}) µg/mL**	**Therapeutic index (in comparison to NO inhibition)**
Fr. 1	>100	>100	>1
Fr. 2	>100	>100	>1
Fr. 3	66.0 ± 5.6	>100	2.0
Fr. 4	76.7 ± 7.0	>100	2.3
Fr. 5	>100	>100	>1
Fr. 6	>100	>100	>1
Fr. 7	>100	>100	>1
Fr. 8	21.7 ± 3.7	>100	>10
Fr. 9	4.4 ± 1.5	16.7 ± 6.8	3.7
Fr. 10	14.8 ± 2.4	93.4 ± 4.8	6.3
Fr. 11	>100	>100	>1
Fr. 12	>100	>100	>1
Fr. 13	12.9 ± 0.1	>100	>7.7
Fr. 14	10.1 ± 1.0	23.5 ± 3.8	2.3
Fr. 15	5.2 ± 0.7	38.1 ± 7.1	7.3
Fr. 16	6.2 ± 0.7	35.2 ± 0.1	5.6
Fr. 17	22.9 ± 1.2	>100	>4.5
Fr. 18	64.5 ± 10.0	>100	>1.5
Fr. 19	9.4 ± 0.1	>100	>10

Results represent mean ± SD (n≥3 individual experiments)

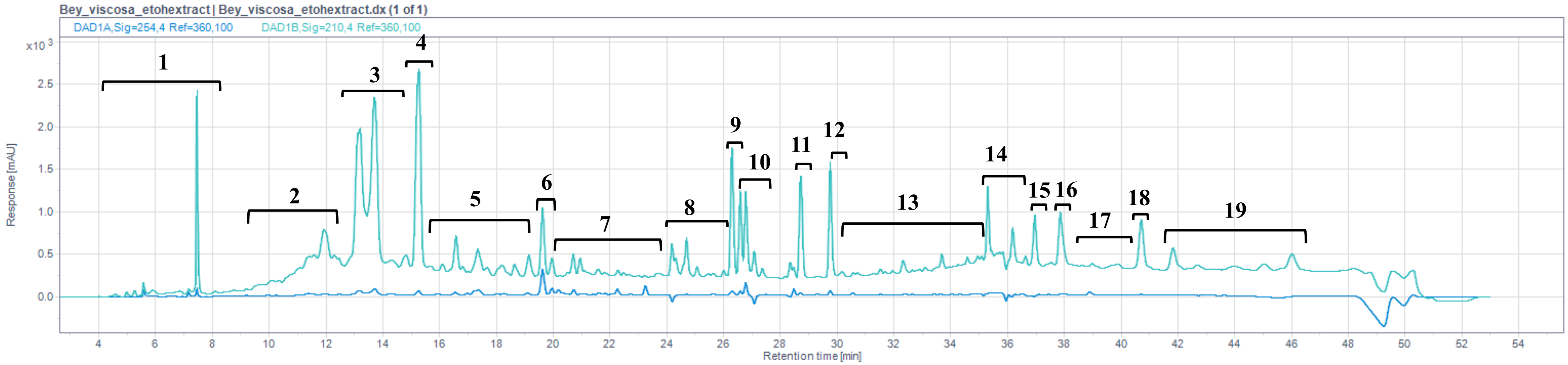

Figure S23. HPLC trace of *Beyeria viscosa* fractions (Fr. 1–19)

HPLC conditions: Semi-preparative Eclipse XDB-C8 Column (5 µm, 9.4 x 250 mm), eluting with 10-100 % MeOH/H_2O (with constant 0.01 % Formic acid modifier) over 30 mins at 2.0 mL/min and held at 100 % MeOH for an additional 15 mins and then equilibrated back to 10 % MeOH within 2 mins and maintained at 10 % MeOH for an additional 6 mins.

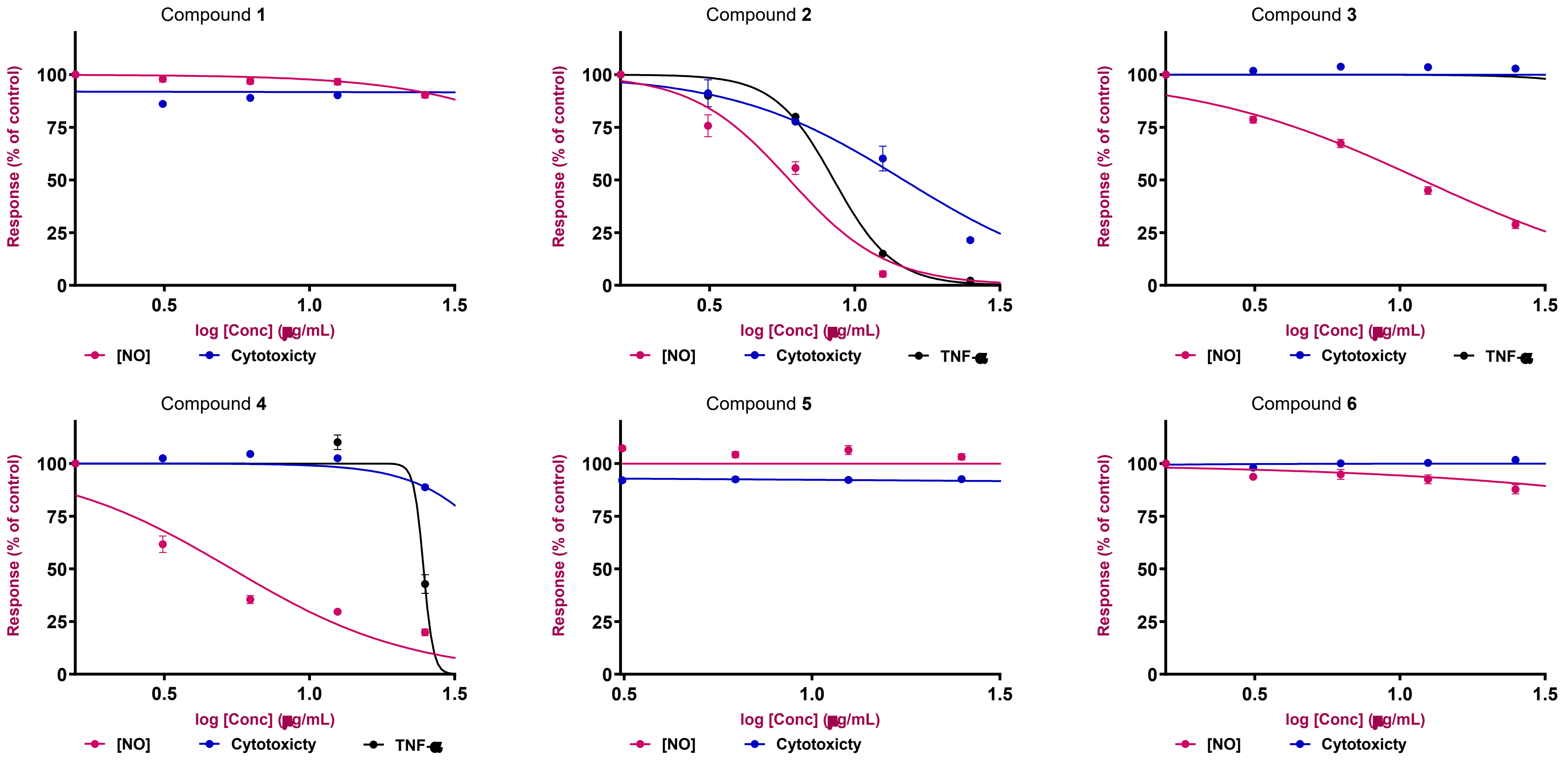

Figure S24. Concentration response curves of compounds **1–6** in RAW 264.7 macrophages showing inhibition of NO production (IC$_{50}$)**,** cell viability (LC$_{50}$), and suppression of TNF-α (IC$_{50}$) under LPS/IFN-γ stimulation. Data are presented as mean ± SD of 3 individual experiments in triplicate. IC$_{50}$ and LC$_{50}$ values were calculated by sigmoidal dose response function and are summarized in in the main text (Table 1).

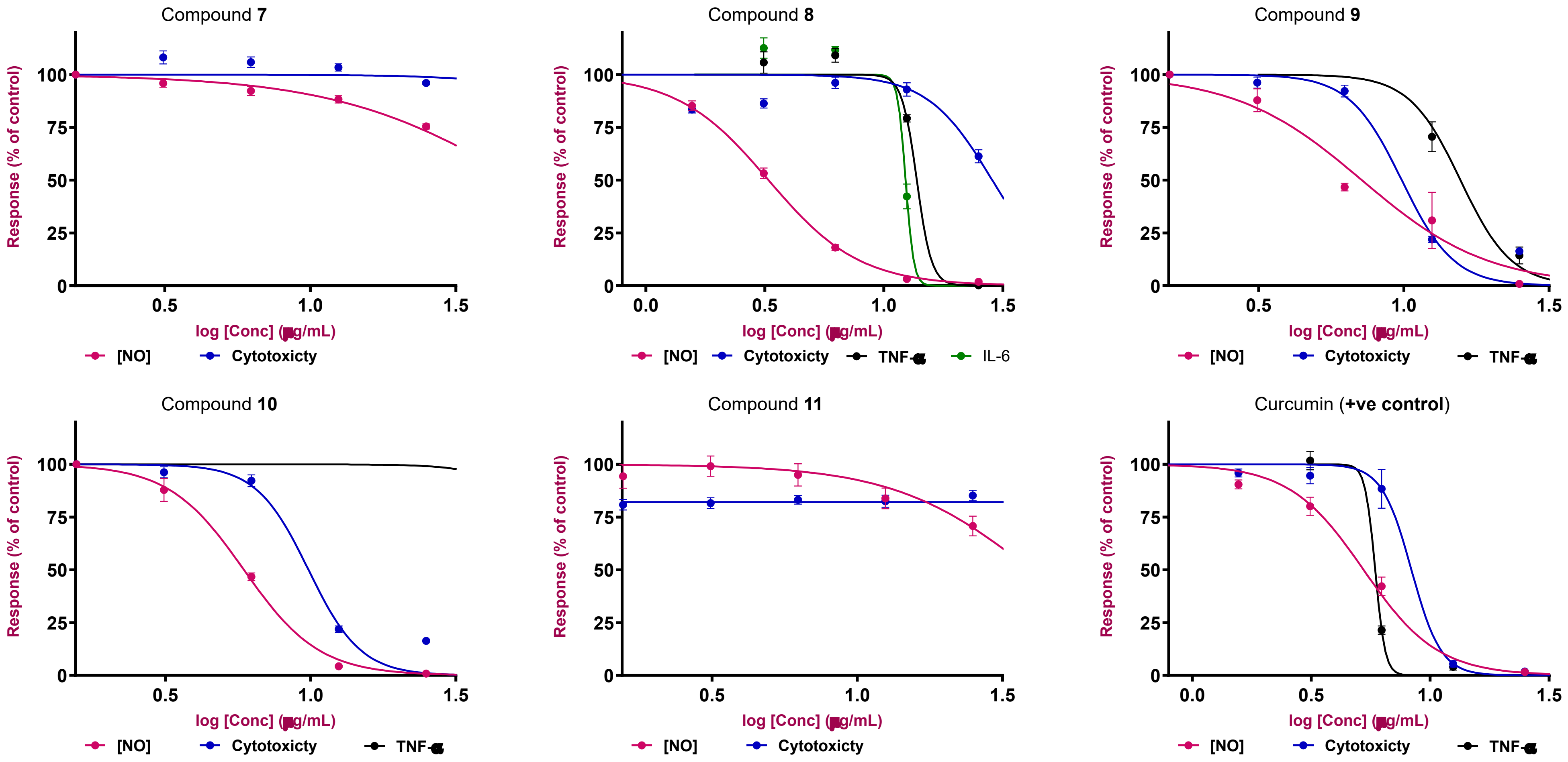

Figure S25. Concentration response curves of compounds **7–11** and curcumin (**+ve control**) in RAW 264.7 macrophages showing inhibition of NO production (IC_{50}), cell viability (LC_{50}), and suppression of TNF-α (IC_{50}) under LPS/IFN-γ stimulation. Data are presented as mean ± SD of 3 individual experiments in triplicate. IC_{50} and LC_{50} values were calculated by sigmoidal dose response function and are summarized in the main text (Table 1).
